# Macronutrient Supplements in Preterm and Small-for-Gestational-Age Animals: A Systematic Review and Meta-analysis

**DOI:** 10.1038/s41598-019-51295-6

**Published:** 2019-10-11

**Authors:** Emma Amissah, Luling Lin, Gregory D Gamble, Caroline A. Crowther, Frank H. Bloomfield, Jane E. Harding

**Affiliations:** Liggins Institute, University of Auckland, Auckland, New Zealand

**Keywords:** Paediatric research, Preclinical research, Outcomes research

## Abstract

Early macronutrient supplementation in preterm and/or small-for-gestational-age (SGA) infants may improve growth but have detrimental effects on later cardio-metabolic health which may be sex-specific. We systematically reviewed the long-term effects of early macronutrient supplementation in preterm and SGA animals and whether these differ by sex. Using Cochrane Neonatal and SYRCLE methodologies we included random or quasi-random studies that allocated non-human mammals to macronutrient supplements or no supplements between birth and weaning and assessed post-weaning outcomes. We used random-effects models to calculate standardized mean differences (SMD) with 95% confidence intervals (CIs). Six studies provided low to very-low-quality evidence that macronutrient supplementation increased weight in juvenile rats (SMD; 95% CI: 2.13; 1.00, 3.25; 1 study, n = 24), increased leptin concentrations in older adults (1.31; 0.12, 2.51; 1 study, n = 14 male rats), but decreased leptin concentrations in young adults (−1.13; −2.21, −0.05; 1 study, n = 16 female rats) and improved spatial learning and memory (qualitative data; 1 study). There was no evidence of sex-specific effects and no overall effect on length, serum lipids, body composition, HOMA-IR, or blood pressure. Macronutrient supplements may affect later growth, metabolism, and neurodevelopment of preterm and SGA animals, but evidence is limited and low quality.

## Introduction

Preterm and small-for-gestational-age infants are at increased risk of postnatal growth restriction, and of adverse short and long-term developmental and health outcomes^1–3^. Postnatal growth restriction results from a complex interplay of several factors but is largely attributed to inadequate nutrition in the form of protein and energy deficits, especially during the early postnatal period^4,5^. Macronutrient supplements are commonly given to infants born small to improve postnatal growth and neurodevelopmental outcomes. However, they may increase the risk of long-term adiposity, metabolic and cardiovascular disease^6^.

Animal studies have supported an association between early life nutrition and long-term risk of metabolic dysfunction^7–10^. Furthermore, some have reported sex-specific differences in the impact of early nutritional interventions on later outcomes. For example, low protein diets in pregnant rats are associated with a higher risk of hypertension in male than in female offspring^7,11,12^. Another study which fed methyl-deficient diets to female rats 3 weeks prior to conception and during the first 5 days of gestation noted sex-specific changes in insulin action in the offspring^13^, while in neonatal lambs, nutritional supplementation with a milk fortifier showed sex-specific effects on later pancreatic function^14^.

However, the basis of these sex differences in response to early life nutritional insults is unclear, and the findings are not consistently observed between different animal models and different species^15^. There is a paucity of data from clinical studies regarding sex differences in the short- and long-term effects of early life nutritional interventions. We aimed to systematically evaluate the available evidence from randomized or quasi-randomized studies on the long-term effects of early macronutrient supplementation in preterm and/or growth restricted or SGA animals, and whether these differ between sexes. The results of this review were intended to inform the design of new clinical trials to improve long-term health outcomes of preterm and SGA infants by providing appropriate sex-specific macronutrient supplements.

## Results

### Study selection

**Figure 1:**
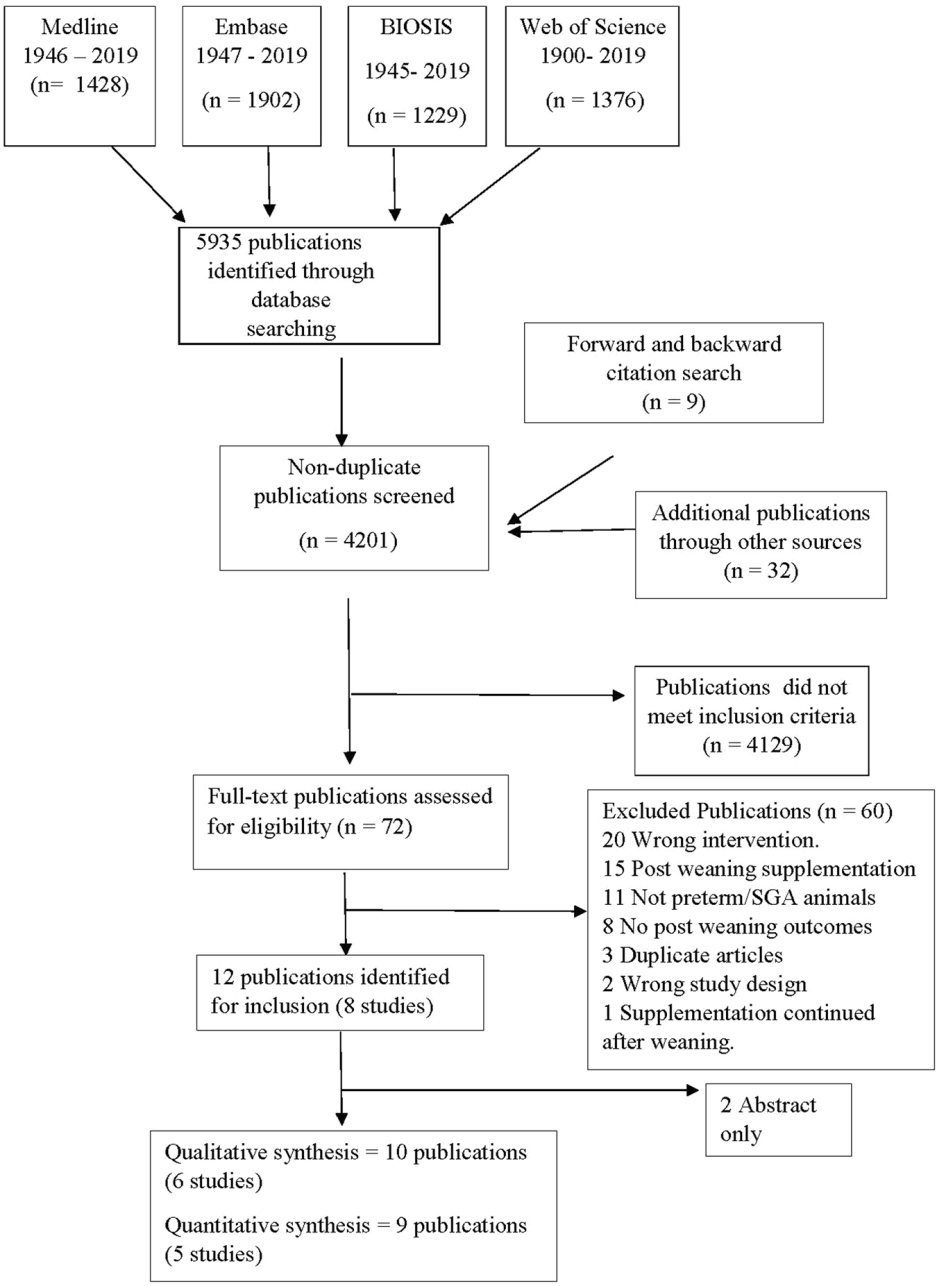
Flowchart of the literature search and study selection process.

The database search yielded 5935 publications, while forward and backward citation search yielded an additional 9, and the search for additional studies suggested by co-authors yielded 32 additional publications. After title and abstract screening, 72 publications met the inclusion criteria and were retrieved for full-text review. Of these, 60 publications were excluded, mostly because they reported the wrong intervention, wrong animal population or lacked post-weaning outcomes (Fig. 1). A total of 12 publications (8 studies) were identified for inclusion in this review^16–27^. Two of these which were available only in abstract form were excluded as they did not report useable data^19,20^. One further study did not report the number of animals per group for each outcome and was excluded from the meta-analysis^23^. Thus, we based the qualitative synthesis on 6 studies and the meta-analyses on 5 studies^16–18,21,22^ (Fig. 1).

### Description of the included studies

Of the six studies included in this review, the authors of five had no conflicts of interest to declare, while one study did not comment on conflicts of interest. Funding was provided by public institutions for all six studies.

The animal populations included Sprague-Dawley rats (3 studies)^18,21,23^, cross-bred piglets (2 studies)^17,22^, and sheep (one study)^16^. Two studies^16,23^ used preterm animals, and four used SGA animals (Table 1). One study used exclusively male rats^18^, another used only female rats^21^, while the remaining studies used animals of both sexes.

**Table 1:** Characteristics of animal studies included in the systematic review. IM = intra-muscular; SD = standard deviation; TG = term gestational length given by authors; HP = high protein; DHA = docosahexaenoic acid; ^#^information was missing or not reported in a useable format in the publication.

Author and year	Species/strain	Preterm/SGA	Birth induction method	Animal sex	Birth weight (kg) (mean ± SD)		Gestational age at birth (TG) (days)	Intervention	Duration of supplementation	Outcomes
					Intervention	Control				
Berry *et al*.^16^	Sheep (n = 60)	Preterm	IM dexamethasone sodium phosphate on days 135 and 136	Males and females	4.84 ± 0.81 (n = 27)	4.75 ± 0.81 (n = 33)	137 (147)	Multicomponent ewe milk supplement	Day 1 to 22 after birth	Growth, metabolic and cardiovascular
Blat *et al*.^17^	Cross-bred pigs (Pietrain x (Large White x Landrace)) (n = 44)	Term SGA	#	Males and females	0·99 ± 0·072 (n = 13)	0.98 ± 0.108 (n = 13)	#	HP formula powder	Day 7 to 28 after birth	Growth, metabolic and genetics
Delamaire *et al*.^18^	Rats Sprague-Dawley (n = 28)	Term SGA	#	Males	#	#	22 (22–23)	HP formula powder	Day 6 to 20 after birth	Growth, metabolic and meal patterns
Qiu *et al*.^21^	Rats Sprague-Dawley (n = 32)	Term SGA	Spontaneous	Females	0.005 ± 0.0004 (n = 16)	0.005 ± 0.0005 (n = 8)	#	Different doses of protein and energy - HP and high energy groups	Birth to 21 days	Growth and metabolic
Sarr *et al*.^22^	Cross-bred pigs (Pietrain x (Large White x Landrace)) (n = 34)	Term SGA	#	Males and females	0.94 ± 0.082 (n = 17)	0.91 ± 0.082 (n = 17)	#	HP formula powder	Day 2 to 28 after birth	Growth, metabolic and genetics
Wang *et al*.^23^	Rats Sprague-Dawley (n = #)	Preterm	Cesarean section	Males and females	#	#	21 (22–23)	Different doses of DHA – sufficient, enriched, excess and deficient groups	Day 1 to 21 after birth	Growth, cognitive learning, and memory

Macronutrient supplementation was done mostly with high protein formulae^17,18,22^, but multi-nutrient^16^ and lipid-based (docosahexaenoic acid, DHA) supplements were also used^23^. Two studies were four-armed (three interventions), with sufficient, excess and enriched DHA groups in one study^23^ and low protein, high protein and high energy groups in another study^21^. The low protein group was excluded from this review. Supplementation commenced between birth and the end of the first postnatal week in all studies and lasted until the end of the species-specific conventional weaning period for all included studies except for one study in sheep where supplementation occurred only during the first two weeks postnatally^16^ (Table 1). The comparator group received standard diet (the composition of which was not well described) in four studies^17,18,21,22^, water as a control supplement in one^16^ and normal saline in one^23^).

None of the included studies provided data for our co-primary outcome measures (cognitive or learning impairment and metabolic risk) or the composite secondary measure of death and impairment. All six studies reported on growth in weight. One study provided data on cardiovascular risk outcomes as blood pressure measures^16^ and five studies contributed data on metabolic outcomes^16–18,21,22^. Only one study assessed the effects of macronutrient supplementation on cognitive and learning impairment and is reported as a narrative as it did not provide data that could be included in the meta-analysis^23^ (Table 1).

### Risk of bias in included studies

Due to poor reporting of methodological details, we judged most of the domains as unclear risk of bias (Supplementary Fig. S1). None of the studies reported on methods used to implement randomization, allocation concealment, blinding, random housing, and random outcome assessment. Using a single characteristic (birth weight), four studies reported similarities between the treatment groups at baseline^16–18,22^. Two studies were judged to have high selective reporting bias due to the authors failing to adequately report specific outcomes^18,21^. Only one study reported the methods and assumptions used in the sample size calculation^17^.

### Effects of macronutrient supplementation versus no supplementation

#### Growth and body composition

**Figure 2:**
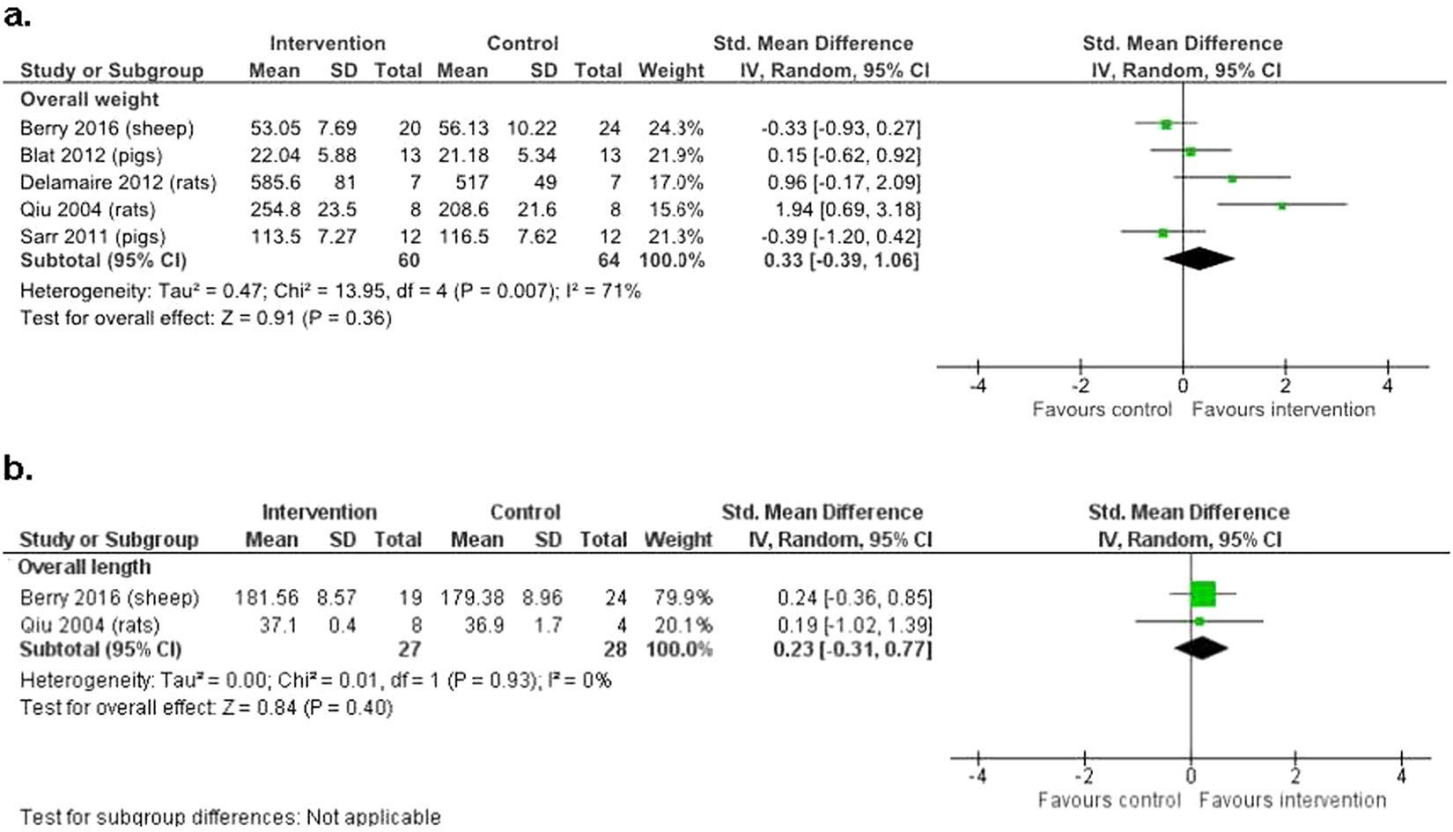
The effect of macronutrient supplementation vs. no supplementation on (**a**) weight and (**b**) length. Forest plots showing standardized mean differences with 95% confidence intervals. We included results of the last time point (oldest age) from each study in the overall summary effect for each outcome.

All six included studies reported post-weaning weight. In the pooled meta-analyses, five studies^16–18,21,22^ of 124 animals (44 sheep, 50 pigs, and 30 rats) contributed weight data while two studies^16,21^ of 55 animals (43 sheep and 12 rats) contributed length data. Overall, there was no evidence of a clear difference between the macronutrient supplemented and unsupplemented groups for weight (SMD 0.33, 95% CI −0.39, 1.06; 5 studies, n = 124 animals, I² = 71%, very low-quality evidence) (Fig. 2a) or length (SMD 0.23, 95% CI −0.31, 0.77; 2 studies, n = 55 animals, I² = 0%, very low-quality evidence) (Fig. 2b). Between-study heterogeneity was substantial among studies contributing data on weight.

One study which was excluded from the meta-analysis reported that pubertal rats born preterm and supplemented with enriched and sufficient amounts of docosahexaenoic fatty acid (DHA) had higher body weights (232.5 ± 4.5 g and 228.4 ± 4.3 g, respectively) compared with unsupplemented pups (206.2 ± 3.9 g)^23^.

**Figure 3:**
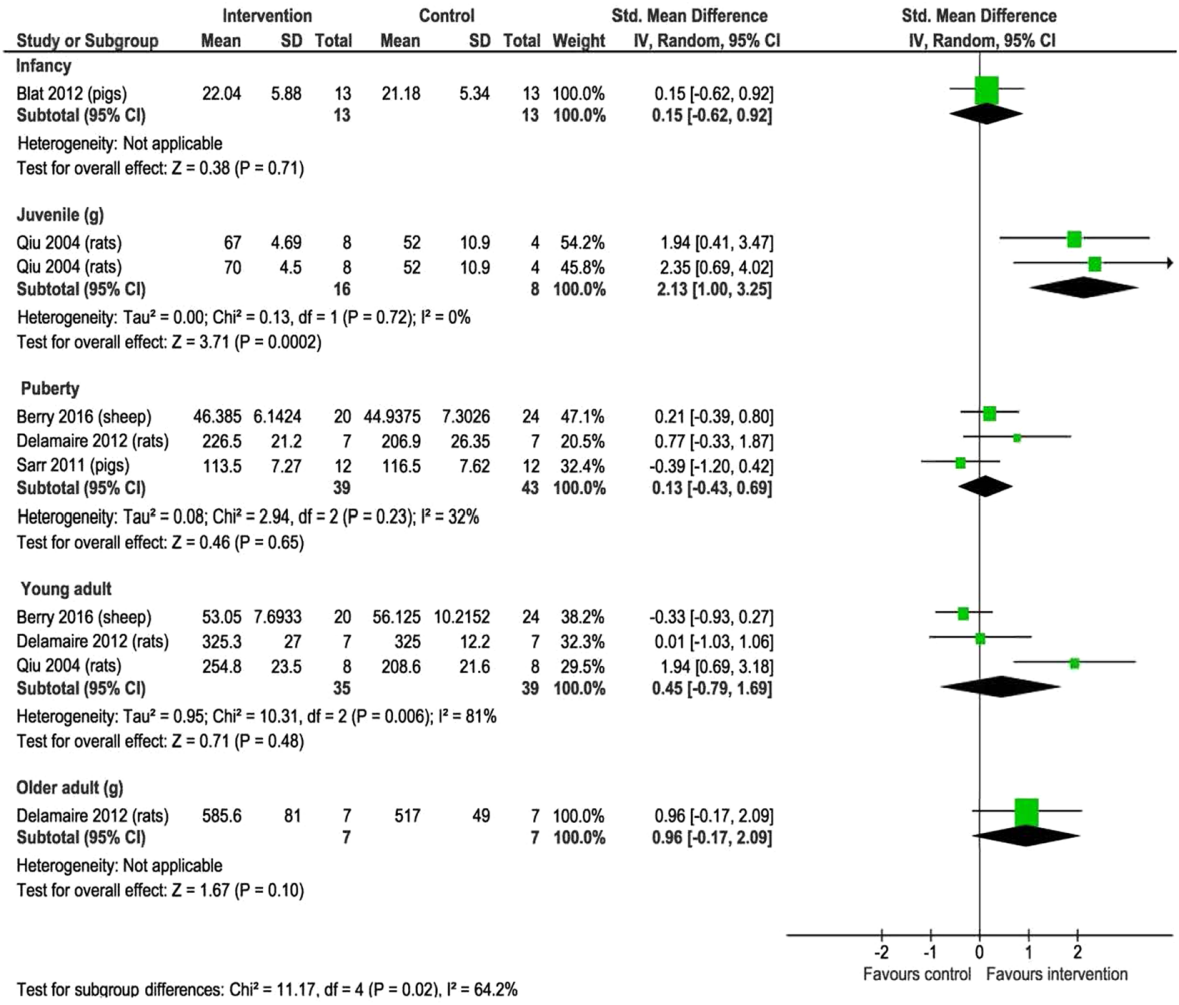
The effect of macronutrient supplementation vs. no supplementation on weight at different ages. Forest plot comparing different age groups with standardized mean differences and 95% confidence intervals.

Subgroup analysis showed that the effect of supplementation was different at different ages (p = 0.02 for interaction). Macronutrient supplementation significantly increased weight in juvenile rats (SMD 2.13, 95% CI 1.00, 3.25; 1 study, n = 24, P = 0.02) but not in other age groups (Fig. 3). Heterogeneity was substantial across the subgroups and within the subgroup of young adults. There were no other significant subgroup differences in weight and length (Supplementary Fig. S2).

#### Metabolic outcomes

**Figure 4:**
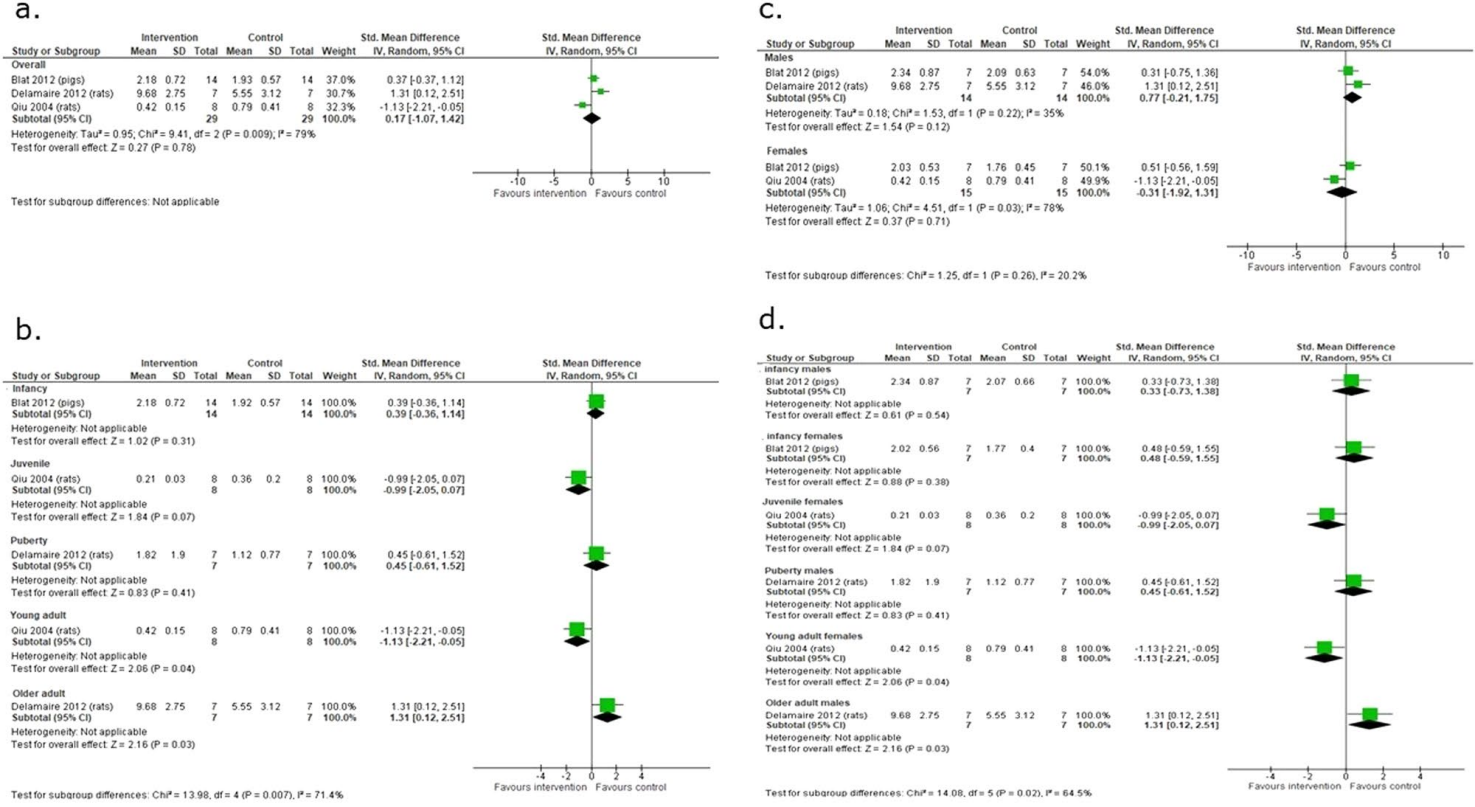
The effect of macronutrient supplementation vs. no supplementation on serum leptin (**a**) overall; (**b**) age subgroups; (**c**) sex subgroups; (**d**) age and sex subgroups. Forest plots showing standardized mean differences and 95% confidence intervals.

Three studies of 58 animals (30 rats and 28 pigs) evaluated the later effects of macronutrient supplementation on serum leptin^17,18,21^. Overall, there was no evidence of a clear difference between the macronutrient supplemented and unsupplemented groups in serum leptin (SMD 0.17 95% CI −1.07 to 1.42; 3 studies, n = 58 animals I² = 79%). However, there were significant differences in the effects of macronutrient supplements on serum leptin concentrations at different ages and in age and sex subgroups, and also significant heterogeneity (age, P = 0.007, I² = 71%, age and sex P = 0.02, I² = 65%). Serum leptin concentrations were reduced in macronutrient supplemented young adult female rats (SMD −1.13; 95% CI; −2.21, −0.05; 1 study, n = 16) but increased in supplemented older adult male rats (SMD 1.31; 95% CI; 0.12, 2.51; 1 study, n = 14) compared with the unsupplemented group (Fig. 4).

**Figure 5:**
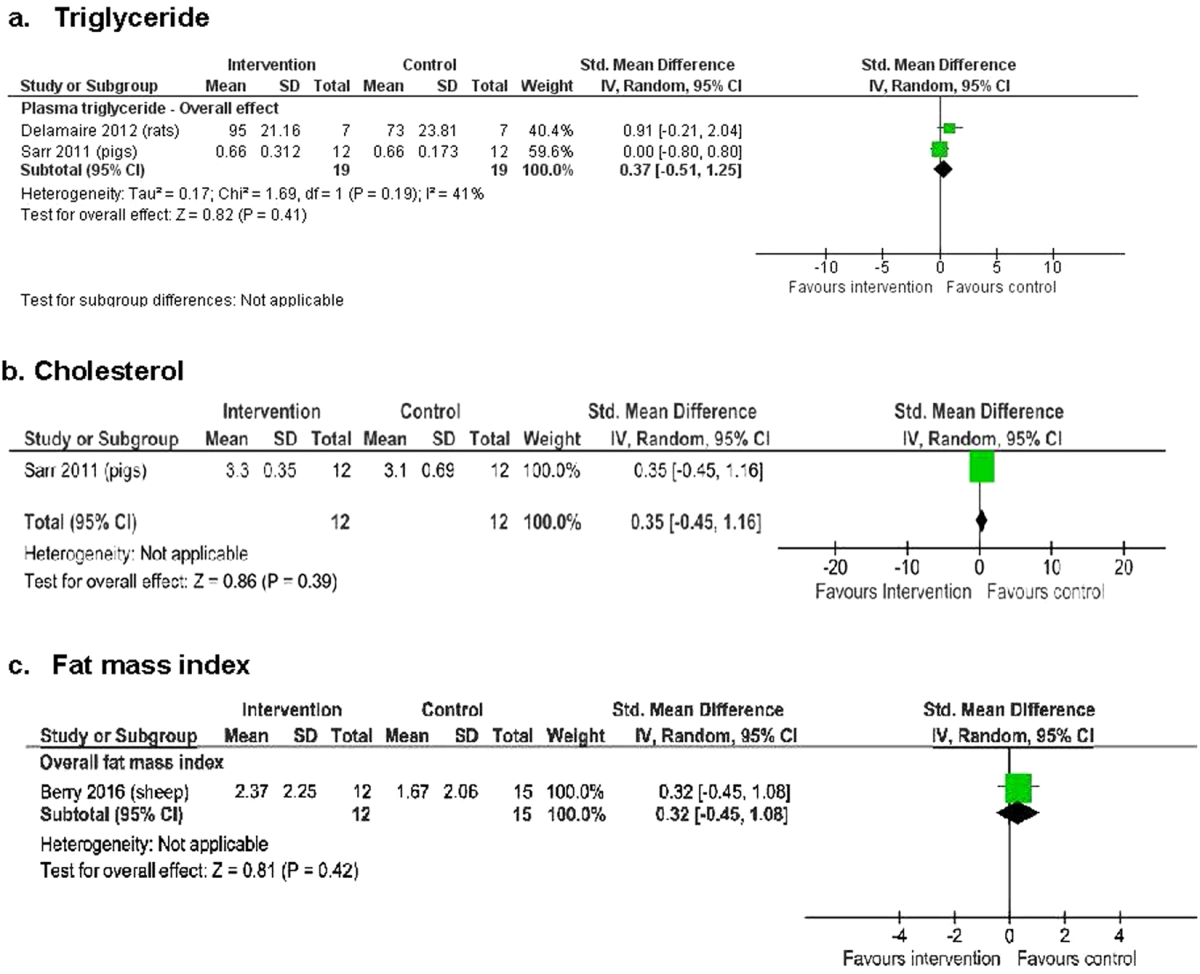
The effect of macronutrient supplementation vs. no supplementation on (**a**) serum triglycerides; (**b**) serum cholesterol; and (**c**) fat mass index. Forest plots showing standardized mean differences with 95% confidence intervals.

**Figure 6:**
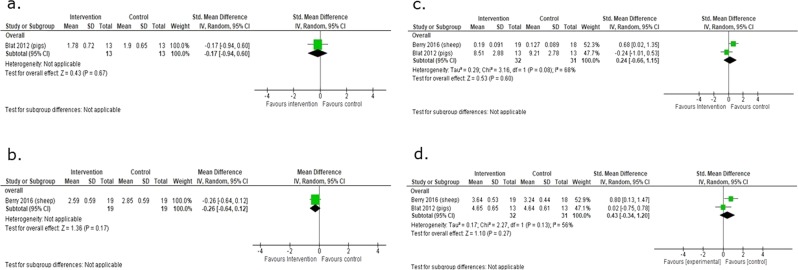
The effect of macronutrient supplementation vs. no supplementation on (**a**) HOMA-IR; (**b**) insulin sensitivity; (**c**) fasting insulin concentrations; and (**d**) fasting plasma glucose concentrations. Forest plots showing standardized mean differences with 95% confidence intervals.

Regarding other metabolic outcomes, two studies of 38 animals (14 rats and 24 pigs) assessed effects on serum lipids^18,22^, while one study of 27 sheep, one study of 26 pigs, one study of 38 sheep, and two studies of 63 animals (37 sheep and 26 pigs) assessed effects on fat mass index [fat mass (kg)/(crown-rump length + hind limb length (m))^2^]^16^, insulin resistance (HOMA-IR)^17^, insulin sensitivity^16^, fasting insulin concentrations and fasting plasma glucose concentrations^16,17^ respectively (Figs 5 and 6). There were no significant differences overall between the macronutrient supplemented and unsupplemented groups for serum lipids (plasma triglycerides: SMD 0.37, 95% CI −0.51 to 1.25; 2 studies, n = 38 animals, I² = 41%^18,22^; total cholesterol: SMD 0.35, 95% CI −0.45 to 1.16; 1 study, n = 24 animals)^22^ or fat mass index (SMD 0.32, 95% CI −0.45 to 1.08; 1 study, n = 27 sheep)^16^ (Fig. 5). There were no differences between groups for HOMA-IR (SMD −0.17, 95% CI −0.94 to 0.60; 1 study, n = 26 pigs, low-quality evidence), insulin sensitivity (SMD −0.26, 95% CI −0.64 to 0.12; 1 study, n = 38 sheep), fasting insulin concentrations (SMD 0.24, 95% CI −0.66 to 1.15; 2 studies, n = 63 sheep, I² = 68%), or fasting plasma glucose concentrations (SMD 0.43, 95% CI −0.34 to 1.20; 2 studies, n = 63 sheep, I² = 56%) (Fig. 6).

There were no other significant subgroup differences observed for any metabolic outcomes except for serum leptin as described above (Supplementary Fig. S3).

#### Blood pressure

**Figure 7:**
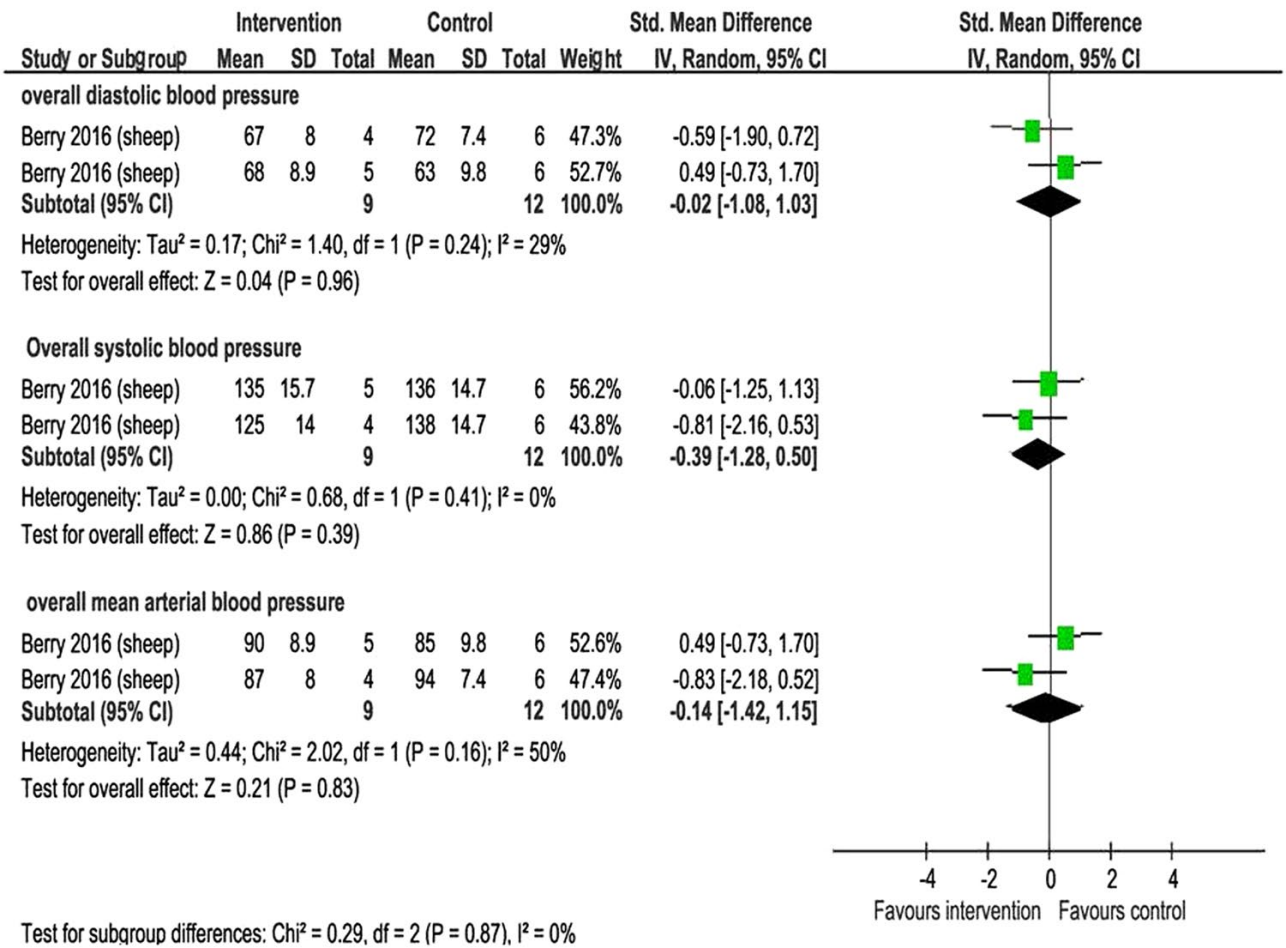
The effect of macronutrient supplementation vs. no supplementation on diastolic, systolic and mean blood pressure. Forest plots showing standardized mean differences with 95% confidence intervals.

One study of 21 sheep evaluated the later effects of macronutrient supplementation on blood pressure^16^. There was no evidence of a clear difference between the macronutrient supplemented and unsupplemented groups in mean arterial (SMD −0.14, 95% CI −1.42 to 1.15; 1 study, n = 21 sheep, I² = 50% very low-quality evidence), diastolic (SMD −0.02, 95% CI −1.08 to 1.03; 1 study, n = 21 sheep) or systolic blood pressure (SMD −0.39, 95% CI −1.28 to 0.50; 1 study, n = 21 sheep) (Fig. 7). There were no significant subgroup differences in the effect of macronutrient supplementation on blood pressure.

#### Cognitive or learning impairment

One study of rats investigated spatial learning and memory^23^. This study found using the Morris water maze test that pubertal pups born preterm and supplemented with sufficient and enriched docosahexaenoic fatty acid (DHA) had improved spatial learning and memory while those supplemented with excess or no DHA had delayed spatial learning and memory. No subgroup analyses were reported.

#### Nutrition

Two studies of 38 animals (14 rats and 24 pigs)^18,22^ assessed the effects of macronutrient supplements on energy intake and one study of 14 rats^18^ assessed effects on appetite. There was no evidence of a clear difference between macronutrient supplemented and unsupplemented animals in energy intake (SMD 0.16, 95% CI −0.48, 0.80; 2 studies, n = 38 animals, I² = 0%) or appetite which was evaluated by feeding time (SMD −0.20, 95% CI −1.25, 0.85; 1 study, n = 14 rats), meal size (SMD 1.10, 95% CI −0.05, 2.25; 1 study, n = 14 rats), speed of ingestion (SMD 0.66, 95% CI −0.43, 1.74; 1 study, n = 14 rats) and inter-meal interval (SMD −0.39, 95% CI −1.45, 0.68; 1 study, n = 14 rats) (Supplementary Figs S4–5).

No data were available to assess the co-primary outcomes of cognitive or learning impairment and metabolic risk, or the secondary outcomes the composite measure of death or impairment and bone growth. We were unable to assess publication bias due to lack of data (<10 included studies) and sensitivity analysis was not performed because the included studies ranged from low to very-low-quality (Supplementary Table S2).

## Discussion

To the best of our knowledge, this is the first study to systematically review and meta-analyse data from studies assessing later effects of macronutrient supplements fed between birth and weaning to animals born small. From six studies using different animal species with low to very-low-quality evidence, macronutrient supplementation of preterm and SGA animals (1) increased weight in juvenile rats but not overall (2) decreased serum leptin concentrations in young adults and increased leptin concentrations in older adults but showed no effect overall (3) had no overall effect on insulin sensitivity, fasting insulin concentrations, fasting plasma glucose concentrations, and mean arterial, systolic or diastolic blood pressure and (4) improved spatial learning and memory. There were insufficient data to reliably assess sex-specific and long-term health effects.

Our meta-analyses showed increases in weight among juvenile rats in the macronutrient supplemented group, but this increase was not seen in adulthood. This may reflect the limited data available, and/or the lack of repeated measurements in the same studies at different ages. However, it is also possible that the shorter term effects of macronutrient supplements on weight were obscured at later ages by the many other factors that influence weight at older ages, including contemporaneous diet, energy expenditure, and genetics.

The observed increase in weight among juvenile rats in the macronutrient supplemented group could be due to increases in fat mass rather than lean mass. One study reported that body composition following macronutrient supplementation was not changed^16^. However, most studies included in this review reported weight alone, without other growth measures such as length/height and body composition. Since body fat rather than total weight is a key predictor of later metabolic risk, and non-invasive measures such as weight for length as an estimate of adiposity and body composition as measured with dual-energy X-ray absorptiometry (DXA) or magnetic resonance imaging (MRI) are now widely available, future nutrition studies should use these parameters rather than body weight alone as key outcomes.

We found no overall effect of macronutrient supplements on serum leptin concentrations, but the effects seen appeared to vary with the age and sex of the animal, being lower in young adult females and higher in older adult males. Leptin is secreted into the circulation by white adipocytes and regulates food intake, body weight, and energy expenditure, amongst other functions^28,29^. Since leptin is influenced by nutritional intake^30^, it has been hypothesized that early nutrition may influence serum leptin concentrations which in turn may alter the risk of obesity in later life^31–34^. In humans and other animals, for instance, leptin intake from mother’s milk or as a supplement during lactation alters serum leptin concentrations in infancy and changes subsequent susceptibility to the development of obesity^35–38^. Interestingly, these effects appear to be sex-specific. For example, prenatal calorie restriction resulted in hypoleptinemia in male and female rats, which enhanced leptin sensitivity and protected against obesity in males, but initiated early leptin resistance in females^39^. The studies included in our review that reported serum leptin concentrations did not perform the experiments in males and females simultaneously, and they compared exposure to a high protein diet with standard diet following prenatal maternal undernutrition. Nonetheless, the limited data included suggest that macronutrient supplements given between birth and weaning may also alter later leptin concentrations in ways that differ by age and sex.

We found no association overall between early macronutrient supplementation and other metabolic outcomes including serum lipids (triglycerides and total cholesterol), fat mass index, HOMA-IR, insulin sensitivity, fasting insulin concentrations, fasting plasma glucose concentrations, and mean arterial, systolic or diastolic blood pressure in animals born small. Although our findings are from limited data, they are consistent with studies of human cohorts born preterm that found no associations between higher protein and energy intakes and serum lipids (triglycerides, HDL cholesterol, and cholesterol) in adults^40^ and systolic or diastolic blood pressure in adolescents^41^. Similarly, at 6 years, a follow-up study of 239 preterm infants randomised to fortified vs. unfortified human milk and preterm formula vs.unfortified human milk, also found no differences in mean arterial, systolic or diastolic blood pressure, and no differences in fat mass index between the groups, although there was a significant increase in fat-free mass index in the unfortified breastmilk group^42^. In contrast, a study using nutrient-enriched diet in term SGA infants beyond the weaning period reported increases in fat mass in later childhood^43^. These findings from randomized trials suggest that early macronutrient supplementation may not be associated with later cardio-metabolic risks, as has been suggested from observational studies^41^. However, of the four studies that reported metabolic outcomes, we noted substantial variation in choice of metabolic outcomes, and this lack of standardization precludes meaningful comparison of data between studies and species.

The only study not included in the meta-analyses investigated cognitive outcomes after supplementation with docosahexaenoic acid (DHA) in preterm rats. It is therefore not possible to distinguish the effects of the macronutrient supplement (fat) from any specific effects of DHA that may or may not apply to supplementation with other lipids. DHA is essential for neural membrane and glial cell development in preterm infants^44^. The study included in this review fed varying doses of DHA to preterm rats^23^ and the authors reported improved spatial learning and memory in preterm pubertal rats fed sufficient (100 mg/kg/d) and enriched doses (300 mg/kg/d) of DHA supplementation, although excess supplementation (800 mg/kg/d) was associated with delayed spatial learning and memory. These findings suggest a dose-dependent effect of DHA on cognitive outcomes. However, findings across randomized trials assessing the cognitive benefits of DHA supplementation in infants born small have been inconsistent, and the optimal intake of DHA for this group of infants is still unknown^45^. A recent systematic review of randomized trials in preterm infants examining the effects of LCPUFA-supplemented formula compared with standard formula found no cognitive benefits of supplementation at 12 and 18 months^46^. However, doses of DHA tested in five of the seven studies included in the review were below or around the median concentration of DHA (~0.3%) in human milk worldwide^47^, and therefore likely to be too low to influence functional outcomes. Daily doses of 1 to 1.5% of total fatty acid as DHA are recommended to emulate *in utero* accretion and to compensate for early deficits occurring between birth and discharge in preterm infants^48,49^.

This review has several strengths. Firstly, the review was designed to address clinically relevant questions, pertinent to the long-term health and well-being of infants born small.

Secondly, to reduce bias, we prepared and registered the protocol for this review *a priori*. Thirdly, we used a systematic process to retrieve, review and extract data for the analysis.

This review also has some limitations. Although our search strategy was comprehensive and devoid of language restrictions, we were able to include only six eligible studies, and no data were available for some important outcomes: co-primary cognitive or learning impairment and metabolic risk; a composite measure of death or impairment, and bone growth. The limited data may be for several reasons. Our inclusion and exclusion criteria may have excluded many studies, and the studies included in the review only followed up the animals for limited periods. Animal studies are particularly important because, unlike human studies, they can be used to investigate adult outcomes after early interventions over shorter timeframes, but in this review, only 3 out of 6 included studies reported outcomes after puberty.

Assessment of the features of cognitive impairment in animal studies can be difficult, and this may have contributed to the lack of data for this important outcome. Infants born small are vulnerable to nutritional deficits and subsequent development of neurocognitive impairments, and nutritional supplements have been introduced into clinical practice in large part to improve cognitive outcomes^50^. Nevertheless, since this is a primary clinical concern, future animal studies of the effects of early nutritional supplements should consider how neurological outcomes might be measured, potentially including imaging to assess brain growth as well as assessment of behaviour and cognitive skills.

Animal studies have not addressed our pre-specified clinically relevant questions concerning sex-specific and long-term effects of early postnatal macronutrient supplementation in infants born small. A systematic review conducted on the same topic in humans yielded 44 studies including 4,812 babies^51,52^. This suggests that the effects of nutritional supplementation have been assessed directly in humans in the absence of data from animal studies. Thus, arguably, answers to our clinical question might be more appropriately sought in long-term follow-up of previously conducted randomized trials in humans rather than in undertaking more animal studies. We found limited data on sex-specific effects which emphasizes the need for adequate power and separate reporting of outcomes for males and females.

We found substantial heterogeneity between studies and subgroups, particularly in the meta-analyses on weight and serum leptin concentrations, where there were most included studies. The heterogeneity could be due to the combination of studies with different animals and interventions, and because of the different mechanisms underlying the experimental growth restriction induced in different studies which may impact differently on the potential for postnatal accelerated growth and/or metabolic outcomes. The appropriateness of combining studies with different types of animals (sheep, rats, and pigs) and interventions in the meta-analysis is debatable, although others have deemed this valid in animal studies^53^. Despite our pre-planned subgroup analyses, the origin of the heterogeneity was unable to be further investigated due to insufficient data. Sensitivity analyses based on study quality to determine the robustness of our findings also was not possible as all the studies were judged to be of low to very-low-quality. It is well-recognized that research methods in animal studies are often inadequately reported^54^, and this was consistent with our findings that none of the included studies reported details on randomization, allocation concealment, random housing, blinding and random outcome assessment as required by the SYRCLE’s risk of bias tool^55^. Randomization, for instance, contributes to the internal validity of a study^56^ and we had planned to include only randomized studies. However, with no information on the randomization techniques used, it was impossible to judge their validity and to draw conclusions about causal relationships from the included studies. The value and credibility of systematic reviews partly depend on the quality of the included studies. Therefore it is important that conduct and reporting of experimental animal studies adheres to the same rigor as randomized controlled trials^57,58^ to reduce bias in results.

## Implications

High-quality animal studies can provide valuable data on aetiology and help to elucidate underlying biological mechanisms of human diseases. In this review, we aimed to assess the long-term and sex-specific effects of macronutrient supplementation in preterm and small-for-gestational-age animals, with the intent to inform the design of new clinical trials to assess sex-specific nutritional supplementation of babies born small. Although we identified a large number of nutritional supplement studies, very few met the eligibility criteria for this review and the data were limited with low to very-low-quality evidence. We found some long-term effects of macronutrient supplements on growth, metabolism, and neurodevelopment, but no sex-specific effects. Future animal studies should adhere to existing guidelines for reporting methodological details, e.g., the ARRIVE guidelines^54^, use clinically relevant nutritional supplement regimes, and assess clinically relevant life-long and sex-specific effects. However, considering the very large number of human studies on this topic, long-term follow-up of these studies may be more appropriate.

## Conclusion

We conclude from this review that long-term sex-specific effects of early macronutrient supplementation remain uncertain. Limited low to very-low-quality evidence shows that macronutrient supplements may increase weight in juveniles, have sex-specific effects on serum leptin concentrations in adults and improve spatial learning and memory in pubertal rats. The preclinical research community should consider developing standardized outcome measures for use in nutritional studies, and any future studies of the effects of early nutritional supplements in preterm or SGA animals should adhere to reporting guidelines and report outcomes pertinent to clinical practice.

## Methods

The methods in this review were pre-specified in a study protocol which is registered with the international prospective register of systematic reviews (PROSPERO) as CRD42018100581 and can be found at https://www.crd.york.ac.uk/prospero/display_record.php?RecordID=100581.

### Search strategy

We searched Embase, Medline, and BIOSIS via OvidSP and Web of Science for studies published from the commencement of the databases to April 19, 2019, with no restrictions on the date of publication, language, and type of study (Supplementary Table S1). We identified relevant MeSH terms in each database’s thesaurus but used the same keywords (listed in Supplementary Table S1) for all searches, and applied a published animal filter^59^. We also searched reference lists of publications identified for inclusion in this review, conducted a forward search using Google Scholar’s “cited by” feature, and emailed researchers familiar with nutritional experiments in animal studies to ask about additional published or ongoing studies meeting our inclusion criteria. We removed duplicates of identified publications using EndNote (version X8.2, Clarivate Analytics, PA, USA) and Covidence [2018 Computer program] (Veritas health innovation, Melbourne, Australia).

### Study Selection

Two reviewers (E.A and L.L) independently screened titles and abstracts and then all available full-text versions of studies identified for inclusion. We resolved discrepancies by discussion or with a third author (J.E.H). We considered for inclusion published and unpublished randomized and quasi-randomized studies involving the provision of supplemental macronutrients to preterm and/or SGA animals (non-human mammals) with the intention of altering long-term growth, developmental or metabolic outcomes. Inclusion criteria were: (i) started supplementation any time between birth and weaning and reported outcomes after weaning; (ii) included any of the following dietary comparisons: (a) any unsupplemented animal feed (the feed may be milk or non-milk based) vs. the same animal feed supplemented; (b) parenteral formulation A versus parenteral formulation B with different macronutrient compositions; (c) maternal milk feeds (mother’s own or donor/foster) versus non-milk based feed of different macronutrient composition; (d) supplemented milk (mother’s own or donor/foster) versus non-milk based feed; (e) enteral feed A versus enteral feed B with different macronutrient type; and (iii) assessed any of our pre-defined outcomes including co-primary cognitive or learning impairment and metabolic risk and secondary outcomes (composite measure of death or impairment, growth, metabolic, cardiovascular, bone, brain and nutritional outcomes) (Supplementary Note 1).

We excluded studies that: (i) utilized supplements other than of macronutrients, e.g., studies reporting the effects of feeding with different amounts of micronutrients (including sodium, potassium, calcium, phosphorous, vitamins, other minerals); (ii) assessed the timing of the introduction of nutrition (early versus delayed feeding); (iii) compared different compositions of the same macronutrient (e.g., different types of lipids or proteins); and (iv) reported none of our pre-specified outcomes (Supplementary Note 1) or no post-weaning outcomes or outcomes involving gastrointestinal development only.

Full-text of one non-English (Chinese) study identified for inclusion in the systematic review was translated using Google scholar.

### Data extraction

We developed a data extraction form prior to two reviewers (E.A and L.L) performing independent data extraction. We extracted data such as general study characteristics (author details, publication year, study design), animal characteristics (species/strain, sex, preterm/SGA birth definition, method of preterm/SGA birth induction, gestational age, birth weight), setting (research centre/farm, season, date of study, inclusion/exclusion criteria, source of funding, conflicts of interest), sample size calculation, intervention and outcomes. We resolved discrepancies by discussion or with a third author (J.E.H). Where data were presented graphically, we extracted it using WebPlotDigitizer 4.1 (https://apps.automeris.io/wpd/).

### Risk of bias analysis

Two reviewers (EA) and (LL) independently assessed risk of bias of individual studies for all ten domains of SYRCLE’s risk of bias tool (sequence generation, baseline characteristics, allocation concealment, random housing, blinding (performance and detection bias), random housing, incomplete outcome data, selective outcome reporting, and other sources of bias). Reviewers did not assess publications of which they were authors. Discrepancies were resolved by discussion or with a third author (JH). We attempted to contact authors of all studies included in this review, as all failed to report important details on randomization, blinding, and on other domains of SYRCLE’s risk of bias. Only two authors responded^16,17^.

### Quality of evidence

We used the Grading of Recommendations Assessment, Development and Evaluation approach (GRADE) for assessment of quality of evidence for key outcomes: cognitive or learning impairment; metabolic risk; a composite measure of death or impairment; weight; length/height; blood pressure; insulin resistance and presented the findings using GRADEpro GDT Guideline Development Tool.

### Statistical analysis

Meta-analyses were performed when at least two studies used the same type of intervention (e.g., high protein diet) and/or outcome measure. We categorized the data by the age of outcome assessment into the following groups: infancy, juvenile, puberty, young and older adult (Supplementary Note 2). Where assessments were carried out at multiple time points within a particular age group, we only used results from the last time point. We performed separate meta-analyses for each age group but included the results of the last time point (oldest age) from each study in the overall summary effect for the outcome^60^. For studies with one control group and more than one treatment group, we compared each intervention being tested with the control group but divided the number of animals in the control group by the number of intervention groups for each comparison to avoid counting control animals more than once^61^. We obtained total data for studies where male and female data were only reported separately for the same intervention and outcome by combining sample sizes, means and standard deviations of males and females in the intervention and control groups using the RevMan calculator [Review Manager Version 5.1 (RevMan, 2014)].

We summarized the results using random effects models as data from the included studies were based on different preterm/SGA animal populations. Because authors used different animals and different scales of measurement for the same outcome, we employed the standardized mean difference with units of standard deviation and 95% confidence intervals (CIs) to estimate effect sizes throughout the meta-analyses. We assessed statistical heterogeneity using the chi² test and the I² statistic and considered an I² measurement of >50% and a P value < 0.10 to indicate substantial heterogeneity (Higgins 2011).

We performed subgroup analyses using Cochran’s Q test and Higgins’ I^2^ to investigate the effect of macronutrient supplementation in preterm/SGA animals of different ages and in each sex. Due to insufficient data we were unable to undertake other pre-planned subgroup analysis in: preterm birth categories (early preterm <88% term gestation vs. late preterm 88% to <92.5% term gestation), size for gestational age of the animal (<25th percentile vs. <10th percentile vs. <3rd percentile), timing of the supplement (from birth vs. from start of enteral feeding vs. in first half of period until weaning vs. in second half of period until weaning), duration of supplementation (≤0.5 vs >0.5 of the period from birth to weaning), type of supplement, (protein vs. carbohydrate vs. lipid vs. multicomponent and their interactions), type of animal feed (milk based vs. non- milk based), and species and groups of species e.g. rodents, ruminants. We had also planned to conduct sensitivity analyses by including only those studies considered to have a low risk of bias for allocation concealment and randomization. However, we were unable to do this as most of the included studies were judged to have an unclear risk of bias. We planned to create funnel plots for the assessment of publication bias, but this was not possible due to insufficient data. We set statistical significance of the effect sizes at a p-value of < 0.05. All analyses were done using Review Manager Version 5.1 (RevMan, 2014).

## Supplementary information

Supplementary material

## Data Availability

The datasets generated during and/or analysed during the current study are available from the corresponding author on reasonable request. Published data are available to approved researchers under the data sharing arrangements provided by the Maternal and Perinatal Central Coordinating Research Hub (CCRH), based at the Liggins Institute, University of Auckland (https://wiki.auckland.ac.nz/researchhub). Metadata, along with instructions for data access, are available at the University of Auckland’s research data repository, Figshare (https://auckland.figshare.com). Data access requests are to be submitted to the Data Access Committee via researchhub@auckland.ac.nz. Data will be shared with researchers who provide a methodologically sound proposal and have appropriate ethical and institutional approval. Researchers must sign and adhere to the Data Access Agreement that includes a commitment to using the data only for the specified proposal, to store data securely and to destroy or return the data after completion of the project. The CCRH reserves the right to charge a fee to cover the costs of making data available if required.
