# Supplementary material for: Macronutrient Supplements in Preterm and Small-for-Gestational-Age Animals: A Systematic Review and Meta-analysis
Source: Sci Rep. 2019 Oct 11;9:14715. doi: 10.1038/s41598-019-51295-6 (PMC6789152; doi:10.1038/s41598-019-51295-6)
Supplement: Supplementary file 1 — Supplementary material [file 41598_2019_51295_MOESM1_ESM.docx]

**SUPPLEMENTARY INFORMATION**

**Macronutrient Supplements in Preterm and Small-for-Gestational-Age Animals: A Systematic Review and Meta-analysis**

**Authors**

Emma Amissah^1^, Luling Lin^1^, Greg Gamble^1^, Caroline A. Crowther^1^, Frank H Bloomfield^1^, Jane E. Harding^1*^

**Institutions**

^1^Liggins Institute, University of Auckland, Auckland, New Zealand

**Corresponding author contact**

*Liggins Institute, University of Auckland, 85 Park Rd, Grafton, Auckland 1023, NZ

Email: [j.harding@auckland.ac.nz](mailto:j.harding@auckland.ac.nz)

Tel: +64 9 9236439

**Supplementary Note 1: List of outcomes**

Primary outcomes:

The co-primary outcomes were cognitive or learning impairment and metabolic risk.

1. Cognitive or learning impairment: is defined as below -1SD on standard developmental or cognitive tests as defined by trialists.

2. Metabolic risk: includes any of the following (all as defined by trialists)

a. Metabolic outcomes

 Elevated plasma triglyceride concentrations

 Reduced high-density lipoprotein (HDL) concentrations

 Elevated low-density lipoprotein (LDL) concentrations

 Elevated fasting plasma glucose concentration

 Increased insulin resistance

 Impaired glucose tolerance

 Increased proportion with type 2 diabetes

b. Growth outcomes

 Increased proportion overweight and obese

 Increased fat mass or fat mass percentage

c. Cardiovascular risk outcomes

 Increased blood pressure (systolic, diastolic, mean)

 Impaired flow-mediated vasodilatation

Secondary outcomes:

1. A composite measure of death or impairment (any of cerebral palsy, blindness, deafness, gross motor dysfunction, psychomotor dysfunction, cognitive or learning impairment).

2. Components of the composite

 Death – early or later death up to the time of follow-up and cause of death.

 Cerebral palsy (any cerebral palsy, as defined by trialists),

 The severity of cerebral palsy (none, mild, moderate, severe – as defined by trialists),

 Cognitive or learning impairment (none, mild (≤ 1 SD below test mean), moderate (≤ 2 SD below test mean), severe (≤ 3 SD below test mean), or as defined by trialists),

 Visual impairment (none, mild, moderate, severe; as defined by trialists),

 Deafness (none, mild, moderate, severe; as defined by trialists),

 Motor dysfunction (none, mild, moderate, severe; as defined by trialist),

 Measures of psychological/behavioral well-being (as defined by trialist),

 Measures of psychomotor dysfunction (e.g., hyperactivity, grip strength or as defined by trialist).

3. Growth outcomes

 Weight (raw data and z scores)

 Length/height (raw data and z scores)

 Head circumference/width (raw data and z scores)

 Weight for length (weight/length, ponderal index, body mass index)

 Postnatal growth restriction as defined by trialist.

 Body composition (fat mass, fat-free mass, lean mass, or other measures as defined by trialists).

4. Cardiovascular risk outcomes

 Blood pressure (systolic, diastolic or mean)

 Flow-mediated vasodilatation

 Measures of sympathetic and parasympathetic tone, e.g. heart rate variability or as defined by trialist.

 Cardiac size and structure

 Measures of vascular stiffness, e.g. pulse wave velocity or as defined by trialist

5. Metabolic outcomes

 Proportion overweight/obese as defined by trialist

 Proportion with type-2 diabetes as defined by trialist

 Plasma triglyceride concentrations

 High-density lipoprotein (HDL) concentrations

 Low-density lipoprotein (LDL) concentrations

 HDL: LDL ratio

 Fasting blood glucose concentrations

 Insulin concentrations

 Insulin resistance as defined by trialist

 Glucose tolerance as defined by trialist.

6. Bone outcomes

 Bone mineral content

 Volumetric bone mineral density

 Number of fractures

7. Brain outcomes

 Whole brain growth, white matter and grey matter volumes, and volumes of individual brain regions

 Brain maturation measured using MRI (white matter tracts, measures of diffusivity, myelination, surface folding)

 Functional brain imaging

 Histological measures of brain structure and maturation

8. Nutrition outcomes

 Altered feeding tolerance

 Altered intake (protein, energy)

 Altered appetite

**Supplementary Note 2: Age categorization of animals**

Age categories were based on the developmental stage of the animal, or as defined by the study. Generally, we used the following definitions: infant was from birth to weaning, juvenile was from weaning to attainment of sexual maturity, puberty was the stage at which sexual organs were functionally developed, young adult was from puberty to when senescent changes could first be detected, and older adult was when senescence was established.

**Species-specific definitions**

**Rats**:

Infancy 0 -21 d

Juvenile 21 – 48d

Puberty 50 – < 63d

Young adult 63 - < 90d

Older adult > 90d

(Etgen & Pfaff, 2010; Mccutcheon & Marinelli, 2009; Sengupta, 2013)

**Piglets**:

Infancy – 0 -5 weeks

Juvenile – > 5 weeks -5 months

Puberty – >5- < 12 months

Young adult – >12 months – <3 years

Older adults – 3 - 4 years

(Poore & Fowden, 2004; Roese & Taylor, 2006; Tur, 2013)

**Sheep:**

Infancy 0–12 weeks

Juvenile 3-5 months

Puberty 5–8 months

Young adult 9–15 months

Older adult > 15 months

(Berry, Jaquiery, Oliver, Harding, & Bloomfield, 2016; Casburn, 2016.)

**References:**

Berry, M. J., Jaquiery, A. L., Oliver, M. H., Harding, J. E., & Bloomfield, F. H. (2016). Neonatal milk supplementation in lambs has persistent effects on growth and metabolic function that differ by sex and gestational age. *The British Journal of Nutrition*, *116*(11), 1912–1925. https://doi.org/10.1017/S0007114516004013

Casburn, G. (2016). *How to tell the age of sheep*. 2.

Etgen, A. M., & Pfaff, D. W. (2010). *Molecular mechanisms of hormone actions on behavior*. Academic Press.

Mccutcheon, J., & Marinelli, M. (2009). Age matters. *The European Journal of Neuroscience*, *29*, 997–1014. https://doi.org/10.1111/j.1460-9568.2009.06648.x

Morise, A., Sève, B., Macé, K., Magliola, C., Le Huërou-Luron, I., & Louveau, I. (2011). Growth, body composition and hormonal status of growing pigs exhibiting a normal or small weight at birth and exposed to a neonatal diet enriched in proteins. *The British Journal of Nutrition*, *105*(10), 1471–1479. https://doi.org/10.1017/S0007114510005386

Poore, K. R., & Fowden, A. L. (2004). The effects of birth weight and postnatal growth patterns on fat depth and plasma leptin concentrations in juvenile and adult pigs. *The Journal of Physiology*, *558*(1), 295–304. https://doi.org/10.1113/jphysiol.2004.061390

Qiu, X., Huang, T., Shen, Z., Deng, H., Ke, Z., Mei, K., & Lai, F. (2004). Effect of different early nutritional interventions on catch-up growth of rats with intrauterine growth retardation. *Zhonghua Er Ke Za Zhi = Chinese Journal of Pediatrics*, *42*(10), 782–786.

Roese, G., & Taylor, G. (2006). *Basic pig husbandry - the weaner*. 6.

Sarr, O., Gondret, F., Jamin, A., Le Huërou-Luron, I., & Louveau, I. (2011). A high-protein neonatal formula induces a temporary reduction of adiposity and changes later adipocyte physiology. *American Journal of Physiology. Regulatory, Integrative and Comparative Physiology*, *300*(2), R387-397. https://doi.org/10.1152/ajpregu.00459.2010

Sengupta, P. (2013). The laboratory rat: relating its age with human’s. *International Journal of Preventive Medicine*, *4*(6), 624–630.

Tur, İ. (2013). General reproductive properties in pigs. *Turk J Vet Anim Sci*, 5.

Wang, Q., Jia, C., Tan, X., Wu, F., Zhong, X., Su, Z., … Cui, Q. (2018). Different concentrations of docosahexanoic acid supplement during lactation result in different outcomes in preterm Sprague-Dawley rats. *Brain Research*, *1678*, 367–373. https://doi.org/10.1016/j.brainres.2017.11.008

**Supplementary Table S1: search methodology**

| Embase <1980 to 2019 April 19> | |
| --- | --- |
| **#** | **Search Statement** |
| 1 | macronutrient/ |
| 2 | ((macronutrient* or macro-nutrient*) adj5 (supplement* or fortif*)).ti,ab,kw. |
| 3 | fortified food/ |
| 4 | ((fortif* or supplement*) adj3 food*).ti,ab,kw. |
| 5 | caloric intake/ |
| 6 | ((calor* or energy) adj3 (intake* or in-take*)).ti,ab,kw. |
| 7 | diet supplementation/ or fat intake/ |
| 8 | ((diet* or nutrition* or fat* or fatty or lipid*) adj2 (supplement* or fortif*)).ti,ab,kw. |
| 9 | ((diet* or nutrition* or fat or fats or fatty or lipid*) adj2 (intake* or in-take*)).ti,ab,kw. |
| 10 | diet* adj2 energy.ti,ab,kw. |
| 11 | omega 3 fatty acid/ or omega 6 fatty acid/ or fish oil/ or medium chain triacylglycerol/ or arachidonic acid/ or docosahexaenoic acid/ or linolenic acid/ or linoleic acid/ |
| 12 | (omega 3 or n-3 fatty or eicosapent* or fish oil* or omega 6 or n-6 fatty or arachidon* or docosahexen* or docosahexaen* or linolenic acid* or linolenate or linoleic or linoleate).ti,ab,kw. |
| 13 | (medium chain adj1 (triacylglycerol* or triglyceride*)).ti,ab,kw. |
| 14 | cod liver oil/ or coconut oil/ or corn oil/ or soybean oil/ or safflower oil/ |
| 15 | ((cod liver or corn or safflower or soy* or coconut) adj1 (oil or oils)).ti,ab,kw. |
| 16 | (((polyunsaturated or poly-unsaturated) adj1 fatty acid*) or PUFA or LCPUFA or icosapent* or timnodon* or lipid* or microlipid* or micro-lipid*).mp. |
| 17 | carbohydrate diet/ or carbohydrate intake/ or exp carbohydrate/ |
| 18 | (carbohydrat* adj1 (supplement* or intake* or in-take* or fortif* or diet*****)).ti,ab,kw. |
| 19 | glucose intake/ or glucan/ or fructose/ |
| 20 | (glucan* or monosaccharide* or disaccharide* or oligosaccharide* or polysaccharide* or polycose or lactose or sucrose or corn syrup or glucose or fructose).mp. |
| 21 | protein intake/ or whey/ |
| 22 | (protein* adj3 (intake* or in-take* or supplement* or fortif* or energy)).ti,ab,kw. |
| 23 | protein hydrolysis/ or whey hydrolysis/ |
| 24 | (whey or protein* hydroly*).ti,ab,kw. |
| 25 | or/1-24 |
| 26 | small for date infant/ or Prematurity/ or Intrauterine growth retardation/ |
| 27 | (small adj3 (baby or babies or date or gestation*)).ti,ab,kw. |
| 28 | ((fetal or foetal or intrauterine or intra-uterine) adj1 growth adj1 (retard* or restrict*)).ti,ab,kw. |
| 29 | ((prematur* or preterm or pre-term) and (infan* or newborn or new-born or neonat* or baby or babies)).ti,ab,kw. |
| 30 | or/26-29 |
| 31 | exp animal experiment/ or exp animal model/ or exp experimental animal/ or exp male animal/ or exp female animal/ or exp juvenile animal/ or animal/ or mammal/ or therian/ or exp monotremate/ or placental mammals/ or exp marsupial/ or Euarchontoglires/ or exp Afrotheria/ or exp Boreoeutheria/ or exp Laurasiatheria/ or exp Xenarthra/ or primate/ or exp Dermoptera/ or exp Glires/ or exp Scandentia/ or Haplorhini/ or exp prosimian/ or simian/ or exp tarsiiform/ or Catarrhini/ or exp Platyrrhini/ or ape/ or exp Cercopithecidae/ or hominid/ or exp hylobatidae/ or exp chimpanzee/ or exp gorilla/ or exp orang utan/ |
| 32 | (animal or animals or shrew or shrews or sorex or araneus or crocidura or russula or european mole or talpa or pup or pups or chiroptera or bat or bats or eptesicus or serotinus or myotis or dasycneme or daubentonii or pipistrelle or pipistrellus or cat or cats or felis or catus or feline or kitten or dog or dogs or canis or canine or canines or puppy or puppies or otter or otters or lutra or badger or badgers or cub or cubs or meles or fitchew or fitch or foumart or foulmart or ferrets or ferret or polecat or polecats or kit or kits or mustela or putorius or weasel or weasels or fox or foxes or vulpes or common seal or phoca or vitulina or grey seal or halichoerus or horse or horses or foal or equus or equine or equidae or donkey or donkeys or mule or mules or pig or pigs or swine or swines or hog or hogs or boar or boars or porcine or piglet or piglets or sus or scrofa or llama or llamas or lama or glama or cria or deer or deers or cervus or elaphus or cow or cows or bos taurus or bos indicus or bovine or bull or bulls or cattle or calf or calves or bison or bisons or sheep or sheeps or ovis aries or ovine or lamb or lambs or mouflon or mouflons or goat or goats or capra or caprine or chamois or rupicapra or leporidae or kid or kids or doeling or doelings or buckling or bucklings or lagomorpha or lagomorph or rabbit or rabbits or oryctolagus or cuniculus or laprine or bunny or bunnies or hares or leveret or leverets or lepus or rodentia or rodent or rodents or murinae or mouse or mice or mus or musculus or murine or woodmouse or apodemus or rat or rats or rattus or norvegicus or guinea pig or guinea pigs or cavia or porcellus or hamster or hamsters or mesocricetus or cricetulus or cricetus or gerbil or gerbils or jird or jirds or meriones or unguiculatus or jerboa or jerboas or jaculus or chinchilla or chinchillas or beaver or beavers or castor fiber or castor canadensis or sciuridae or squirrel or squirrels or sciurus or chipmunk or chipmunks or marmot or marmots or marmota or suslik or susliks or spermophilus or cynomys or cottonrat or cottonrats or sigmodon or vole or voles or microtus or myodes or glareolus or primate or primates or prosimian or prosimians or lemur or lemurs or lemuridae or loris or bush baby or bush babies or bushbaby or bushbabies or galago or galagos or anthropoidea or anthropoids or simian or simians or monkey or monkeys or marmoset or marmosets or callithrix or cebuella or tamarin or tamarins or saguinus or leontopithecus or squirrel monkey or squirrel monkeys or saimiri or night monkey or night monkeys or owl monkey or owl monkeys or douroucoulis or aotus or spider monkey or spider monkeys or ateles or baboon or baboons or papio or rhesus monkey or macaque or macaca or mulatta or cynomolgus or fascicularis or green monkey or green monkeys or chlorocebus or vervet or vervets or pygerythrus or hominoidea or ape or apes or hylobatidae or gibbon or gibbons or siamang or siamangs or nomascus or symphalangus or hominidae or orangutan or orangutans or pongo or chimpanzee or chimpanzees or pan troglodytes or bonobo or bonobos or pan paniscus or gorilla or gorillas or troglodytes).ti,ab. |
| 33 | 31 or 32 |
| 34 | 33 not human/ |
| 35 | 25 and 30 and 34 |

| Embase Classic <1947 to 1979> | |
| --- | --- |
| **#** | **Search Statement** |
| 1 | macronutrient/ or caloric intake/ |
| 2 | ((calor* or energy) adj3 (intake* or in-take*)).ti,ab,kw. |
| 3 | ((macronutrient* or macro-nutrient*) adj5 (supplement* or fortif*)).ti,ab,kw. |
| 4 | ((fortif* or supplement*) adj3 food*).ti,ab,kw. |
| 5 | diet supplementation/ or fat intake/ |
| 6 | diet* adj2 energy.ti,ab,kw. |
| 7 | ((diet* or nutrition* or fat* or fatty or lipid*) adj2 (intake* or in-take*)).ti,ab,kw. |
| 8 | ((diet* or nutrition* or fat* or fatty or lipid*) adj2 (supplement* or fortif*)).ti,ab,kw. |
| 9 | omega 3 fatty acid/ or omega 6 fatty acid/ or fish oil/ or medium chain triacylglycerol/ or arachidonic acid/ or docosahexaenoic acid/ or linolenic acid/ or linoleic acid/ |
| 10 | (omega 6 or n-6 fatty or omega 3 or n-3 fatty or eicosapent* or fish oil* or arachidon* or docosahexen* or docosahexaen* or linolenic acid* or linolenate or linoleic or linoleate).ti,ab,kw. |
| 11 | (medium chain adj1 (triacylglycerol* or triglyceride*)).ti,ab,kw. |
| 12 | coconut oil/ or cod liver oil/ or corn oil/ or safflower oil/ or soybean oil/ |
| 13 | ((cod liver or corn or safflower or soy* or coconut) adj1 (oil or oils)).ti,ab,kw. |
| 14 | (((polyunsaturated or poly-unsaturated) adj1 fatty acid*) or PUFA or LCPUFA or icosapent* or timnodon* or lipid* or microlipid* or micro-lipid*).mp. |
| 15 | carbohydrate diet/ or carbohydrate intake/ or exp carbohydrate/ |
| 16 | (carbohydrat* adj1 (supplement* or intake* or in-take* or fortif* or diet*)).ti,ab,kw. |
| 17 | glucose intake/ or glucan/ or fructose/ |
| 18 | (glucan* or monosaccharide* or disaccharide* or oligosaccharide* or polysaccharide* or polycose or lactose or sucrose or corn syrup or glucose or fructose).mp. |
| 19 | protein intake/ or whey/ or protein hydrolysis/ |
| 20 | (protein* adj3 (intake* or in-take* or supplement* or fortif* or energy)).ti,ab,kw. |
| 21 | (whey or protein* hydroly*).ti,ab,kw. |
| 22 | 1 or 2 or 3 or 4 or 5 or 6 or 7 or 8 or 9 or 10 or 11 or 12 or 13 or 14 or 15 or 16 or 17 or 18 or 19 or 20 or 21 |
| 23 | small for date infant/ or intrauterine growth retardation/ |
| 24 | prematurity/ |
| 25 | (small adj3 (baby or babies or date or gestation*)).mp. |
| 26 | ((fetal or foetal or intrauterine or intra-uterine) adj1 growth adj1 (retard* or restrict*)).ti,ab,kw. |
| 27 | ((prematur* or preterm or pre-term) and (infan* or newborn or new-born or neonat* or baby or babies)).ti,ab,kw. |
| 28 | 23 or 24 or 25 or 26 or 27 |
| 29 | exp animal experiment/ or exp animal model/ or exp experimental animal/ or exp male animal/ or exp female animal/ or exp juvenile animal/ or animal/ or mammal/ or therian/ or exp monotremate/ or placental mammals/ or exp marsupial/ or Euarchontoglires/ or exp Afrotheria/ or exp Boreoeutheria/ or exp Laurasiatheria/ or exp Xenarthra/ or primate/ or exp Dermoptera/ or exp Glires/ or exp Scandentia/ or Haplorhini/ or exp prosimian/ or simian/ or exp tarsiiform/ or Catarrhini/ or exp Platyrrhini/ or ape/ or exp Cercopithecidae/ or hominid/ or exp hylobatidae/ or exp chimpanzee/ or exp gorilla/ or exp orang utan/ |
| 30 | (animal or animals or shrew or shrews or sorex or araneus or crocidura or russula or european mole or talpa or pup or pups or chiroptera or bat or bats or eptesicus or serotinus or myotis or dasycneme or daubentonii or pipistrelle or pipistrellus or cat or cats or felis or catus or feline or kitten or dog or dogs or canis or canine or canines or puppy or otter or otters or lutra or badger or badgers or cub or cubs or meles or fitchew or fitch or foumart or foulmart or ferrets or ferret or polecat or polecats or kit or kits or mustela or putorius or weasel or weasels or fox or foxes or vulpes or common seal or phoca or vitulina or grey seal or halichoerus or horse or horses or foal or equus or equine or equidae or donkey or donkeys or mule or mules or pig or pigs or swine or swines or hog or hogs or boar or boars or porcine or piglet or piglets or sus or scrofa or llama or llamas or lama or glama or cria or deer or deers or fawn or cervus or elaphus or cow or cows or bos taurus or bos indicus or bovine or bull or bulls or cattle or calf or calves or bison or bisons or sheep or sheeps or ovis aries or ovine or lamb or lambs or mouflon or mouflons or goat or goats or capra or caprine or chamois or rupicapra or leporidae or kid or kids or doeling or buckling or lagomorpha or lagomorph or rabbit or rabbits or oryctolagus or cuniculus or laprine or bunny or bunnies or hares or leveret or leverets or lepus or rodentia or rodent or rodents or murinae or mouse or mice or mus or musculus or murine or woodmouse or apodemus or rat or rats or rattus or norvegicus or guinea pig or guinea pigs or cavia or porcellus or hamster or hamsters or mesocricetus or cricetulus or cricetus or gerbil or gerbils or jird or jirds or meriones or unguiculatus or jerboa or jerboas or jaculus or chinchilla or chinchillas or beaver or beavers or castor fiber or castor canadensis or sciuridae or squirrel or squirrels or sciurus or chipmunk or chipmunks or marmot or marmots or marmota or suslik or susliks or spermophilus or cynomys or cottonrat or cottonrats or sigmodon or vole or voles or microtus or myodes or glareolus or primate or primates or prosimian or prosimians or lemur or lemurs or lemuridae or loris or bush baby or bush babies or bushbaby or bushbabies or galago or galagos or anthropoidea or anthropoids or simian or simians or monkey or monkeys or marmoset or marmosets or callithrix or cebuella or tamarin or tamarins or saguinus or leontopithecus or squirrel monkey or squirrel monkeys or saimiri or night monkey or night monkeys or owl monkey or owl monkeys or douroucoulis or aotus or spider monkey or spider monkeys or ateles or baboon or baboons or papio or rhesus monkey or macaque or macaca or mulatta or cynomolgus or fascicularis or green monkey or green monkeys or chlorocebus or vervet or vervets or pygerythrus or hominoidea or ape or apes or hylobatidae or gibbon or gibbons or siamang or siamangs or nomascus or symphalangus or hominidae or orangutan or orangutans or pongo or chimpanzee or chimpanzees or pan troglodytes or bonobo or bonobos or pan paniscus or gorilla or gorillas or troglodytes).ti,ab. |
| 31 | 29 or 30 |
| 32 | 31 not human/ |
| 33 | 22 and 28 and 32 |

| BIOSIS Previews <1969 to 2019 April 19> | |
| --- | --- |
| **#** | **Search Statement** |
| 1 | ((macronutrient* or macro-nutrient*) adj5 (supplement* or fortif*)).mp. |
| 2 | ((fortif* or supplement*) adj3 food*).mp. |
| 3 | ((diet* or nutrition* or fat* or fatty or lipid*) adj2 (intake* or in-take*)).mp. |
| 4 | ((diet* or nutrition* or fat* or fatty or lipid*) adj2 (supplement* or fortif*)).mp. |
| 5 | ((calor* or energy) adj3 (intake* or in-take*)).mp. |
| 6 | diet* adj2 energy.mp. |
| 7 | (protein* adj3 (intake* or in-take* or supplement* or fortif* or energy)).mp. |
| 8 | (whey or protein* hydroly*).mp. |
| 9 | (carbohydrat* adj1 (diet* or supplement* or intake* or in-take* or fortif*)).mp. |
| 10 | (glucan* or monosaccharide* or disaccharide* or oligosaccharide* or polysaccharide* or polycose or lactose or sucrose or corn syrup or glucose or fructose).mp. |
| 11 | (omega 6 or n-6 fatty or omega 3 or n-3 fatty or eicosapent* or fish oil* or arachidon* or docosahexen* or docosahexaen* or linolenic acid* or linolenate or linoleic or linoleate).mp. |
| 12 | ((cod liver or corn or safflower or soy* or coconut) adj1 (oil or oils)).mp. |
| 13 | (((polyunsaturated or poly-unsaturated) adj1 fatty acid*) or PUFA or LCPUFA or icosapent* or timnodon* or lipid* or microlipid* or micro-lipid*).mp. |
| 14 | (medium chain adj1 (triacylglycerol* or triglyceride*)).mp. |
| 15 | or/1-14 |
| 16 | (small adj3 (baby or babies or date or gestation*)).mp. |
| 17 | ((fetal or foetal or intrauterine or intra-uterine) adj1 growth adj1 (retard* or restrict*)).mp. |
| 18 | ((prematur* or preterm or pre-term) and (infan* or newborn or new-born or neonat* or baby or babies)).ti,ab,kw. |
| 19 | or/16-18 |
| 20 | (animal or animals or shrew or shrews or sorex or araneus or crocidura or russula or european mole or talpa or pup or pups or chiroptera or bat or bats or eptesicus or serotinus or myotis or dasycneme or daubentonii or pipistrelle or pipistrellus or cat or cats or felis or catus or feline or kitten or dog or dogs or canis or canine or canines or puppy or otter or otters or lutra or badger or badgers or cub or cubs or meles or fitchew or fitch or foumart or foulmart or ferrets or ferret or polecat or polecats or kit or kits or mustela or putorius or weasel or weasels or fox or foxes or vulpes or common seal or phoca or vitulina or grey seal or halichoerus or horse or horses or foal or equus or equine or equidae or donkey or donkeys or mule or mules or pig or pigs or swine or swines or hog or hogs or boar or boars or porcine or piglet or piglets or sus or scrofa or llama or llamas or lama or glama or cria or deer or deers or fawn or cervus or elaphus or cow or cows or bos taurus or bos indicus or bovine or bull or bulls or cattle or calf or calves or bison or bisons or sheep or sheeps or ovis aries or ovine or lamb or lambs or mouflon or mouflons or goat or goats or capra or caprine or chamois or rupicapra or leporidae or kid or kids or doeling or buckling or lagomorpha or lagomorph or rabbit or rabbits or oryctolagus or cuniculus or laprine or bunny or bunnies or hares or leveret or leverets or lepus or rodentia or rodent or rodents or murinae or mouse or mice or mus or musculus or murine or woodmouse or apodemus or rat or rats or rattus or norvegicus or guinea pig or guinea pigs or cavia or porcellus or hamster or hamsters or mesocricetus or cricetulus or cricetus or gerbil or gerbils or jird or jirds or meriones or unguiculatus or jerboa or jerboas or jaculus or chinchilla or chinchillas or beaver or beavers or castor fiber or castor canadensis or sciuridae or squirrel or squirrels or sciurus or chipmunk or chipmunks or marmot or marmots or marmota or suslik or susliks or spermophilus or cynomys or cottonrat or cottonrats or sigmodon or vole or voles or microtus or myodes or glareolus or primate or primates or prosimian or prosimians or lemur or lemurs or lemuridae or loris or bush baby or bush babies or bushbaby or bushbabies or galago or galagos or anthropoidea or anthropoids or simian or simians or monkey or monkeys or marmoset or marmosets or callithrix or cebuella or tamarin or tamarins or saguinus or leontopithecus or squirrel monkey or squirrel monkeys or saimiri or night monkey or night monkeys or owl monkey or owl monkeys or douroucoulis or aotus or spider monkey or spider monkeys or ateles or baboon or baboons or papio or rhesus monkey or macaque or macaca or mulatta or cynomolgus or fascicularis or green monkey or green monkeys or chlorocebus or vervet or vervets or pygerythrus or hominoidea or ape or apes or hylobatidae or gibbon or gibbons or siamang or siamangs or nomascus or symphalangus or hominidae or orangutan or orangutans or pongo or chimpanzee or chimpanzees or pan troglodytes or bonobo or bonobos or pan paniscus or gorilla or gorillas or troglodytes).ti,ab. |
| 21 | 20 not human/ |
| 22 | 15 and 19 and 21 |
| BIOSIS Previews Archive <1945 to 1968> | |
| **#** | **Search Statement** |
| 1 | ((macronutrient* or macro-nutrient*) adj5 (supplement* or fortif*)).mp. |
| 2 | ((fortif* or supplement*) adj3 food*).mp. |
| 3 | ((diet* or nutrition* or fat* or fatty or lipid*) adj2 (intake* or in-take*)).mp. |
| 4 | ((diet* or nutrition* or fat* or fatty or lipid*) adj2 (supplement* or fortif*)).mp. |
| 5 | ((calor* or energy) adj3 (intake* or in-take*)).mp. |
| 6 | diet* adj2 energy.mp. |
| 7 | (protein* adj3 (intake* or in-take* or supplement* or fortif* or energy)).mp. |
| 8 | (whey or protein* hydroly*).mp. |
| 9 | (carbohydrat* adj1 (diet* or supplement* or intake* or in-take* or fortif*)).mp. |
| 10 | (glucan* or monosaccharide* or disaccharide* or oligosaccharide* or polysaccharide* or polycose or lactose or sucrose or corn syrup or glucose or fructose).mp. |
| 11 | (omega 6 or n-6 fatty or omega 3 or n-3 fatty or eicosapent* or fish oil* or arachidon* or docosahexen* or docosahexaen* or linolenic acid* or linolenate or linoleic or linoleate).mp. |
| 12 | ((cod liver or corn or safflower or soy* or coconut) adj1 (oil or oils)).mp. |
| 13 | (((polyunsaturated or poly-unsaturated) adj1 fatty acid*) or PUFA or LCPUFA or icosapent* or timnodon* or lipid* or microlipid* or micro-lipid*).mp. |
| 14 | (medium chain adj1 (triacylglycerol* or triglyceride*)).mp. |
| 15 | or/1-14 |
| 16 | (small adj3 (baby or babies or date or gestation*)).mp. |
| 17 | ((fetal or foetal or intrauterine or intra-uterine) adj1 growth adj1 (retard* or restrict*)).mp. |
| 18 | ((prematur* or preterm or pre-term) and (infan* or newborn or new-born or neonat* or baby or babies)).ti,ab,kw. |
| 19 | or/16-18 |
| 20 | (animal or animals or shrew or shrews or sorex or araneus or crocidura or russula or european mole or talpa or pup or pups or chiroptera or bat or bats or eptesicus or serotinus or myotis or dasycneme or daubentonii or pipistrelle or pipistrellus or cat or cats or felis or catus or feline or kitten or dog or dogs or canis or canine or canines or puppy or otter or otters or lutra or badger or badgers or cub or cubs or meles or fitchew or fitch or foumart or foulmart or ferrets or ferret or polecat or polecats or kit or kits or mustela or putorius or weasel or weasels or fox or foxes or vulpes or common seal or phoca or vitulina or grey seal or halichoerus or horse or horses or foal or equus or equine or equidae or donkey or donkeys or mule or mules or pig or pigs or swine or swines or hog or hogs or boar or boars or porcine or piglet or piglets or sus or scrofa or llama or llamas or lama or glama or cria or deer or deers or fawn or cervus or elaphus or cow or cows or bos taurus or bos indicus or bovine or bull or bulls or cattle or calf or calves or bison or bisons or sheep or sheeps or ovis aries or ovine or lamb or lambs or mouflon or mouflons or goat or goats or capra or caprine or chamois or rupicapra or leporidae or kid or kids or doeling or buckling or lagomorpha or lagomorph or rabbit or rabbits or oryctolagus or cuniculus or laprine or bunny or bunnies or hares or leveret or leverets or lepus or rodentia or rodent or rodents or murinae or mouse or mice or mus or musculus or murine or woodmouse or apodemus or rat or rats or rattus or norvegicus or guinea pig or guinea pigs or cavia or porcellus or hamster or hamsters or mesocricetus or cricetulus or cricetus or gerbil or gerbils or jird or jirds or meriones or unguiculatus or jerboa or jerboas or jaculus or chinchilla or chinchillas or beaver or beavers or castor fiber or castor canadensis or sciuridae or squirrel or squirrels or sciurus or chipmunk or chipmunks or marmot or marmots or marmota or suslik or susliks or spermophilus or cynomys or cottonrat or cottonrats or sigmodon or vole or voles or microtus or myodes or glareolus or primate or primates or prosimian or prosimians or lemur or lemurs or lemuridae or loris or bush baby or bush babies or bushbaby or bushbabies or galago or galagos or anthropoidea or anthropoids or simian or simians or monkey or monkeys or marmoset or marmosets or callithrix or cebuella or tamarin or tamarins or saguinus or leontopithecus or squirrel monkey or squirrel monkeys or saimiri or night monkey or night monkeys or owl monkey or owl monkeys or douroucoulis or aotus or spider monkey or spider monkeys or ateles or baboon or baboons or papio or rhesus monkey or macaque or macaca or mulatta or cynomolgus or fascicularis or green monkey or green monkeys or chlorocebus or vervet or vervets or pygerythrus or hominoidea or ape or apes or hylobatidae or gibbon or gibbons or siamang or siamangs or nomascus or symphalangus or hominidae or orangutan or orangutans or pongo or chimpanzee or chimpanzees or pan troglodytes or bonobo or bonobos or pan paniscus or gorilla or gorillas or troglodytes).ti,ab. |
| 21 | 20 not human/ |
| 22 | 15 and 19 and 21 |

| Ovid MEDLINE (R) 1946 – April 19 2019 | |
| --- | --- |
| **#** | **Search Statement** |
| 1 | (protein* adj3 (intake* or in-take* or supplement* or fortif* or energy)).ti,ab,kw. |
| 2 | ((macronutrient* or macro-nutrient*) adj5 (supplement* or fortif*)).mp. |
| 3 | Food, Fortified/ |
| 4 | ((fortif* or supplement*) adj3 food*).ti,ab,kw. |
| 5 | Dietary Supplements/ |
| 6 | ((diet* or nutrition* or fat* or fatty or lipid*) adj2 (supplement* or fortif*)).ti,ab,kw. |
| 7 | ((diet* or nutrition* or fat* or fatty or lipid*) adj2 (intake* or in-take*)).ti,ab,kw. |
| 8 | omega 3 fatty acid/ or omega 6 fatty acid/ or fish oil/ or medium chain triacylglycerol/ or arachidonic acid/ or docosahexaenoic acid/ or linolenic acid/ or linoleic acid/ |
| 9 | (omega 6 or n-6 fatty or omega 3 or n-3 fatty or eicosapent* or fish oil* or arachidon* or docosahexen* or docosahexaen* or linolenic acid* or linolenate or linoleic or linoleate).ti,ab,kw. |
| 10 | (medium chain adj1 (triacylglycerol* or triglyceride*)).ti,ab,kw. |
| 11 | coconut oil/ or cod liver oil/ or corn oil/ or safflower oil/ or soybean oil/ |
| 12 | ((cod liver or corn or safflower or soy* or coconut) adj1 (oil or oils)).ti,ab,kw. |
| 13 | (((polyunsaturated or poly-unsaturated) adj1 fatty acid*) or PUFA or LCPUFA or icosapent* or timnodon* or lipid* or microlipid* or micro-lipid*).mp. |
| 14 | Dietary Carbohydrates/ or exp Carbohydrates/ |
| 15 | (carbohydrat* adj1 (diet* or supplement* or intake* or in-take* or fortif*)).ti,ab,kw. |
| 16 | exp Glucans/ or glucose/ or fructose/ |
| 17 | (glucan* or monosaccharide* or disaccharide* or oligosaccharide* or polysaccharide* or polycose or lactose or sucrose or corn syrup or glucose fructose).mp. |
| 18 | dietary proteins/ or Whey/ |
| 19 | (whey or protein* hydroly*).ti,ab,kw. |
| 20 | (protein* adj3 (intake* or in-take* or supplement* or fortif* or energy)).ti,ab,kw. |
| 21 | Energy Intake/ |
| 22 | ((calor* or energy) adj3 (intake* or in-take*)).ti,ab,kw. |
| 23 | diet* adj2 energy.ti,ab,kw. |
| 24 | or/2-23 |
| 25 | infant, small for gestational age/ or infant, premature/ or infant, extremely premature/ |
| 26 | Fetal Growth Retardation/ |
| 27 | (small adj3 (baby or babies or date or gestation*)).mp. |
| 28 | ((fetal or foetal or intrauterine or intra-uterine) adj1 growth adj1 (retard* or restrict*)).ti,ab,kw. |
| 29 | ((prematur* or preterm or pre-term) and (infan* or newborn or new-born or neonat* or baby or babies)).ti,ab,kw. |
| 30 | or/25-29 |
| 31 | exp animal experiment/ or exp animal model/ or exp experimental animal/ or exp male animal/ or exp female animal/ or exp juvenile animal/ or animal/ or mammal/ or therian/ or exp monotremate/ or placental mammals/ or exp marsupial/ or Euarchontoglires/ or exp Afrotheria/ or exp Boreoeutheria/ or exp Laurasiatheria/ or exp Xenarthra/ or primate/ or exp Dermoptera/ or exp Glires/ or exp Scandentia/ or Haplorhini/ or exp prosimian/ or simian/ or exp tarsiiform/ or Catarrhini/ or exp Platyrrhini/ or ape/ or exp Cercopithecidae/ or hominid/ or exp hylobatidae/ or exp chimpanzee/ or exp gorilla/ or exp orang utan/ |
| 32 | (animal or animals or shrew or shrews or sorex or araneus or crocidura or russula or european mole or talpa or pup or pups or chiroptera or bat or bats or eptesicus or serotinus or myotis or dasycneme or daubentonii or pipistrelle or pipistrellus or cat or cats or felis or catus or feline or kitten or dog or dogs or canis or canine or canines or puppy or otter or otters or lutra or badger or badgers or cub or cubs or meles or fitchew or fitch or foumart or foulmart or ferrets or ferret or polecat or polecats or kit or kits or mustela or putorius or weasel or weasels or fox or foxes or vulpes or common seal or phoca or vitulina or grey seal or halichoerus or horse or horses or foal or equus or equine or equidae or donkey or donkeys or mule or mules or pig or pigs or swine or swines or hog or hogs or boar or boars or porcine or piglet or piglets or sus or scrofa or llama or llamas or lama or glama or cria or deer or deers or fawn or cervus or elaphus or cow or cows or bos taurus or bos indicus or bovine or bull or bulls or cattle or calf or calves or bison or bisons or sheep or sheeps or ovis aries or ovine or lamb or lambs or mouflon or mouflons or goat or goats or capra or caprine or chamois or rupicapra or leporidae or kid or kids or doeling or buckling or lagomorpha or lagomorph or rabbit or rabbits or oryctolagus or cuniculus or laprine or bunny or bunnies or hares or leveret or leverets or lepus or rodentia or rodent or rodents or murinae or mouse or mice or mus or musculus or murine or woodmouse or apodemus or rat or rats or rattus or norvegicus or guinea pig or guinea pigs or cavia or porcellus or hamster or hamsters or mesocricetus or cricetulus or cricetus or gerbil or gerbils or jird or jirds or meriones or unguiculatus or jerboa or jerboas or jaculus or chinchilla or chinchillas or beaver or beavers or castor fiber or castor canadensis or sciuridae or squirrel or squirrels or sciurus or chipmunk or chipmunks or marmot or marmots or marmota or suslik or susliks or spermophilus or cynomys or cottonrat or cottonrats or sigmodon or vole or voles or microtus or myodes or glareolus or primate or primates or prosimian or prosimians or lemur or lemurs or lemuridae or loris or bush baby or bush babies or bushbaby or bushbabies or galago or galagos or anthropoidea or anthropoids or simian or simians or monkey or monkeys or marmoset or marmosets or callithrix or cebuella or tamarin or tamarins or saguinus or leontopithecus or squirrel monkey or squirrel monkeys or saimiri or night monkey or night monkeys or owl monkey or owl monkeys or douroucoulis or aotus or spider monkey or spider monkeys or ateles or baboon or baboons or papio or rhesus monkey or macaque or macaca or mulatta or cynomolgus or fascicularis or green monkey or green monkeys or chlorocebus or vervet or vervets or pygerythrus or hominoidea or ape or apes or hylobatidae or gibbon or gibbons or siamang or siamangs or nomascus or symphalangus or hominidae or orangutan or orangutans or pongo or chimpanzee or chimpanzees or pan troglodytes or bonobo or bonobos or pan paniscus or gorilla or gorillas or troglodytes).ti,ab. |
| 33 | 31 or 32 |
| 34 | 33 not human/ |
| 35 | 24 and 30 and 34 |
|  |  |
|  | Web of Science |
| # 1 | TS=(animal or animals or shrew or shrews or sorex or araneus or crocidura or russula or european mole or talpa or pup or pups or chiroptera or bat or bats or eptesicus or serotinus or myotis or dasycneme or daubentonii or pipistrelle or pipistrellus or cat or cats or felis or catus or feline or kitten or dog or dogs or canis or canine or canines or puppy or otter or otters or lutra or badger or badgers or cub or cubs or meles or fitchew or fitch or foumart or foulmart or ferrets or ferret or polecat or polecats or kit or kits or mustela or putorius or weasel or weasels or fox or foxes or vulpes or common seal or phoca or vitulina or grey seal or halichoerus or horse or horses or foal or equus or equine or equidae or donkey or donkeys or mule or mules or pig or pigs or swine or swines or hog or hogs or boar or boars or porcine or piglet or piglets or sus or scrofa or llama or llamas or lama or glama or cria or deer or deers or fawn or cervus or elaphus or cow or cows or bos taurus or bos indicus or bovine or bull or bulls or cattle or calf or calves or bison or bisons or sheep or sheeps or ovis aries or ovine or lamb or lambs or mouflon or mouflons or goat or goats or capra or caprine or chamois or rupicapra or leporidae or kid or kids or doeling or buckling or lagomorpha or lagomorph or rabbit or rabbits or oryctolagus or cuniculus or laprine or bunny or bunnies or hares or leveret or leverets or lepus or rodentia or rodent or rodents or murinae or mouse or mice or mus or musculus or murine or woodmouse or apodemus or rat or rats or rattus or norvegicus or guinea pig or guinea pigs or cavia or porcellus or hamster or hamsters or mesocricetus or cricetulus or cricetus or gerbil or gerbils or jird or jirds or meriones or unguiculatus or jerboa or jerboas or jaculus or chinchilla or chinchillas or beaver or beavers or castor fiber or castor canadensis or sciuridae or squirrel or squirrels or sciurus or chipmunk or chipmunks or marmot or marmots or marmota or suslik or susliks or spermophilus or cynomys or cottonrat or cottonrats or sigmodon or vole or voles or microtus or myodes or glareolus or primate or primates or prosimian or prosimians or lemur or lemurs or lemuridae or loris or bush baby or bush babies or bushbaby or bushbabies or galago or galagos or anthropoidea or anthropoids or simian or simians or monkey or monkeys or marmoset or marmosets or callithrix or cebuella or tamarin or tamarins or saguinus or leontopithecus or squirrel monkey or squirrel monkeys or saimiri or night monkey or night monkeys or owl monkey or owl monkeys or douroucoulis or aotus or spider monkey or spider monkeys or ateles or baboon or baboons or papio or rhesus monkey or macaque or macaca or mulatta or cynomolgus or fascicularis or green monkey or green monkeys or chlorocebus or vervet or vervets or pygerythrus or hominoidea or ape or apes or hylobatidae or gibbon or gibbons or siamang or siamangs or nomascus or symphalangus or hominidae or orangutan or orangutans or pongo or chimpanzee or chimpanzees or pan troglodytes or bonobo or bonobos or pan paniscus or gorilla or gorillas or troglodytes).ti,ab. |
| # 2 | TS=("macronutrient supplement*" OR "macronutrient fortif*" OR caloric intake OR "energy intake*" OR "food fortif*" OR food* fortified OR fortified food* OR "food supplement*" OR "diet supplement*" OR "diet intake*" OR "diet fortif*"OR "nutritional supplement*" OR "nutritional fortif*" OR "nutritional intake*" OR "protein supplement*" OR protein intake OR "protein-energy" OR "protein fortif*" OR "protein hydroly*" OR "whey hydroly*" OR "carbohydrate supplement*" OR carbohydrate intake OR "carbohydrate fortif*" OR glucan* OR monosaccharide* OR disaccharide* OR oligosaccharide* OR polysaccharide* OR polycose OR lactose OR sucrose OR "corn syrup" OR fructose OR glucose OR fat intake OR "fat supplement*" OR "fat fortif*" OR "lipid intake*" OR "lipid supplement*" OR "lipid fortifi*" OR "medium chain triglyceride*" OR "medium chain triacylglycerol*" OR microlipid OR PUFA OR LCPUFA OR "linolenic acid*" OR linolenate OR linoleic OR linoleate OR arachidon* OR docosahexen* OR docosahexaen* OR eicosapent* OR timnodonic OR icosapentaenoic OR "fatty omega-3" OR "Omega-3 Fatty" OR "Omega-6 fatty" OR "N-3 Fatty" OR "N-6 Fatty" OR "Fish oil*" OR "Cod Liver oil*" OR "corn oil*" OR "safflower oil*" OR "soy oil*" OR "coconut oil*" OR "Fatty Acid*" OR Polyunsaturated) |
| # 3 | TS=( "small infant*" OR "small baby" OR "small babies" OR "small for date" OR "small for gestational age" OR "fetal growth retard*" OR "foetal growth retard*" OR "fetal growth restrict*" OR "foetal growth restrict*" OR "intrauterine growth retardat*" OR "intra-uterine growth retardat*" OR "intrauterine growth restrict*" OR "intra-uterine growth restrict*" OR Preterm OR premature* OR pre-term ) |
| # 4 | TS = ( human*) |
| #5 | #1 NOT #4 |
|  | #5 AND #3 AND #2 - 1261 |

#

# Supplementary Table S2. Summary of findings table for macronutrient supplementation vs. no supplementation in preterm and small-for-gestational age animals

| Outcomes | **Anticipated absolute effects^*^** (95% CI) | | Relative effect (95% CI) | № of participants  (studies) | Certainty of the evidence (GRADE) | Comments |
| --- | --- | --- | --- | --- | --- | --- |
|  | **Risk with no supplementation** | **Risk with Macronutrient supplementation** |  |  |  |  |
| Cognitive/learning impairment | No data | - | - | - | - | - |
| Metabolic risk | No data | - | - | - | - |  |
| Composite measure of death and impairment | No data | - | - | - | - | - |
| Growth: weight | - | SMD **0.33 higher** (0.39 lower to 1.06 higher) | - | 124 (5 RCTs) | ⨁◯◯◯ VERY LOW ^a,b,c^ |  |
| Growth: length | - | SMD **0.23 higher** (0.31 lower to 0.77 higher) | - | 55 (2 RCTs) | ⨁◯◯◯ VERY LOW ^a,c^ |  |
| HOMA Insulin Resistance (HOMA-IR) | - | SMD **0.17 higher** (0.94 lower to 0.6 higher) | - | 26 (1 RCT) | ⨁⨁◯◯ LOW ^a,c^ |  |
| Blood pressure: mean arterial blood pressure | - | SMD **0.14 higher** (1.42 lower to 1.15 higher) | - | 21 (1 RCT) | ⨁◯◯◯ VERY LOW ^a,c,d^ |  |
| ***The risk in the intervention group** (and its 95% confidence interval) is based on the assumed risk in the comparison group and the **relative effect** of the intervention (and its 95% CI).   **CI:** Confidence interval; **SMD:** Standardised mean difference | | | | | | |
| **GRADE Working Group grades of evidence** **High certainty:** We are very confident that the true effect lies close to that of the estimate of the effect **Moderate certainty:** We are moderately confident in the effect estimate: The true effect is likely to be close to the estimate of the effect, but there is a possibility that it is substantially different **Low certainty:** Our confidence in the effect estimate is limited: The true effect may be substantially different from the estimate of the effect **Very low certainty:** We have very little confidence in the effect estimate: The true effect is likely to be substantially different from the estimate of effect | | | | | | |

**Explanations**

a. All studies lacked methodological details making it impossible to adequately judge risk of bias. Downgraded one level.

b. Substantial heterogeneity among studies estimating the population mean difference. Downgraded one level.

c. Few animals and wide confidence intervals. Downgraded two levels.

d. Moderate heterogeneity. Downgraded one level

**Supplementary Figure S1. Review authors’ judgements of the ten individual items used in assessing risk of bias for each included study. Expressed as a) percentages in graph and b) a summary.**

**a.**

**
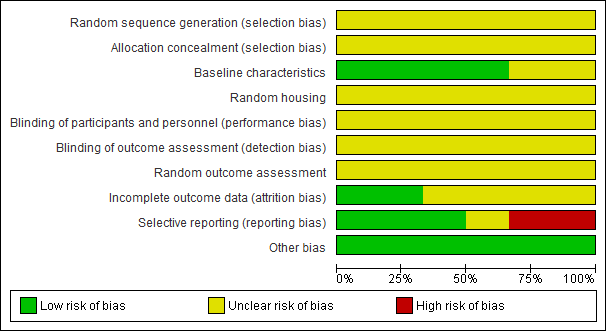
**

**b.**

**
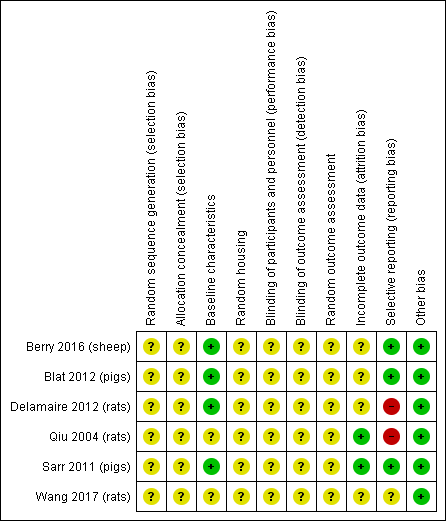

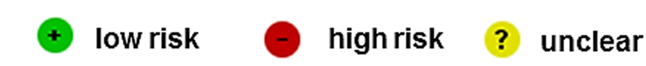
**

# Forest Plots

Review: Macronutrient supplements vs. no supplements in preterm and small-for-gestational age animals.

**Supplementary Figure S2. The effect of macronutrient supplementation vs. no supplementation for weight in a) sex, b) age and sex subgroups, and for length in c) age, d) sex, and e) age and sex subgroups.**

**a.**


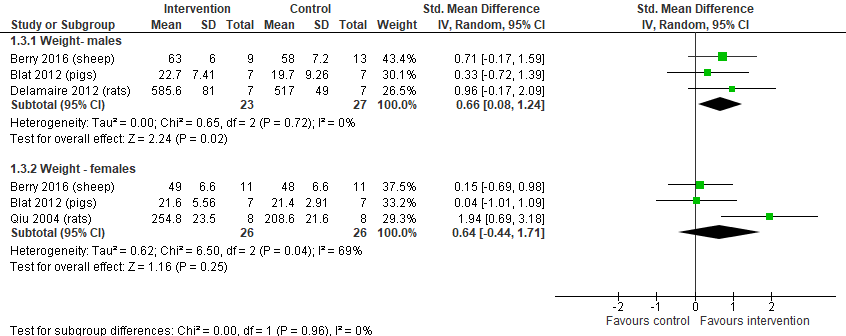


**b.**


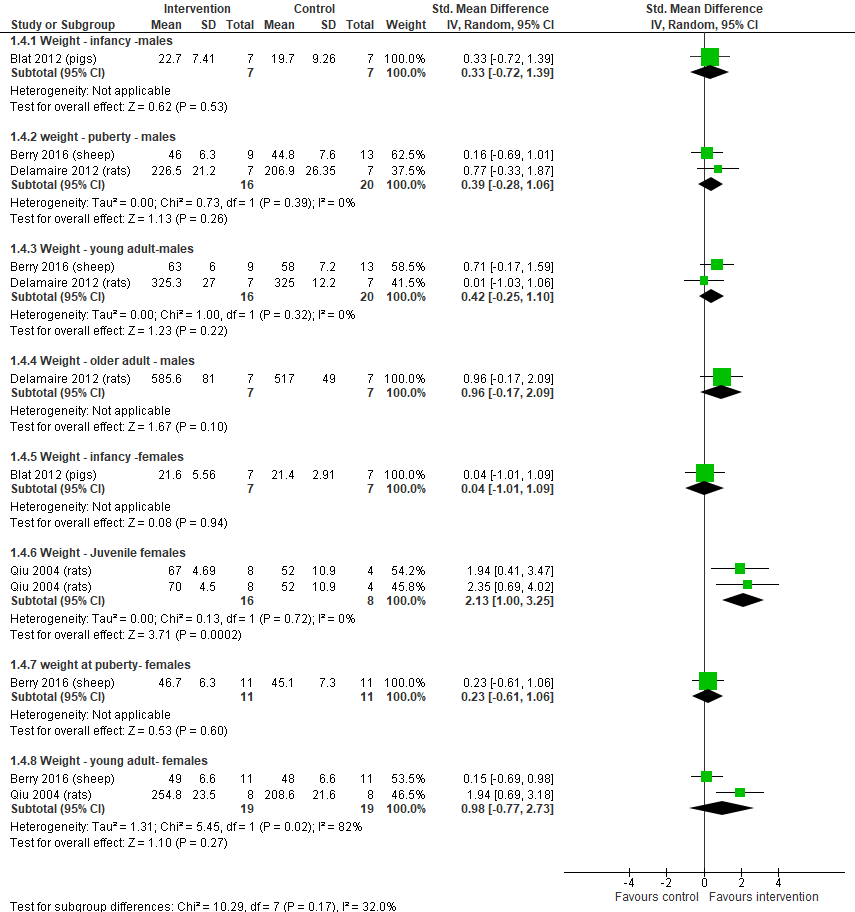


**c.**


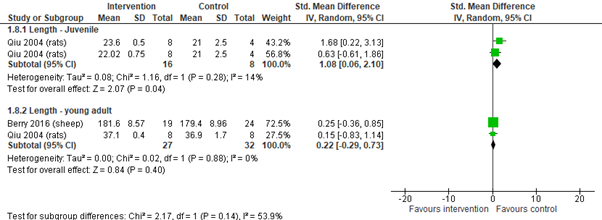


**d.**


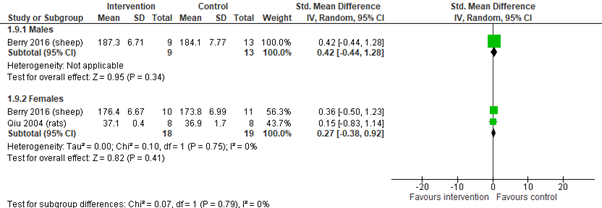


**e.**


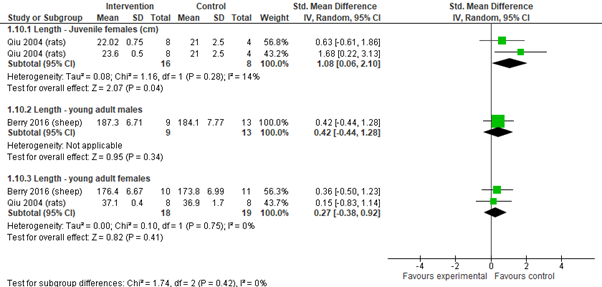


**Forest plots comparing standardized mean differences and 95% confidence intervals**

**Supplementary Figure S3. The effect of macronutrient supplementation vs. no supplementation for: fasting plasma glucose concentrations in a) age b) sex c) age and sex subgroups; insulin sensitivity in d) sex e) age and sex subgroups; and fasting insulin concentrations in f) age, g) sex, h) age and sex subgroups.**

**Fasting plasma glucose**


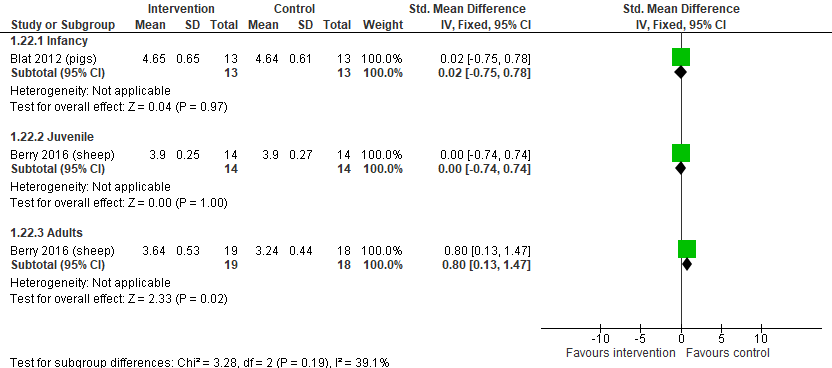


# b.
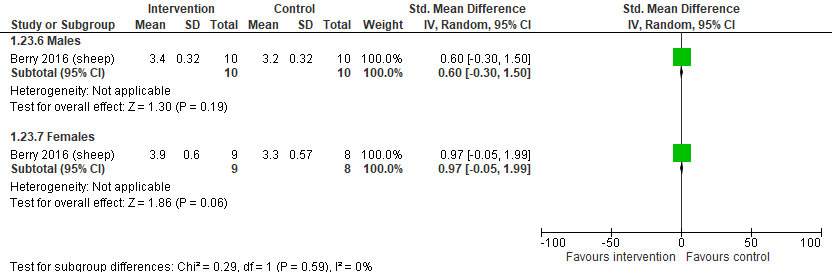


# c.


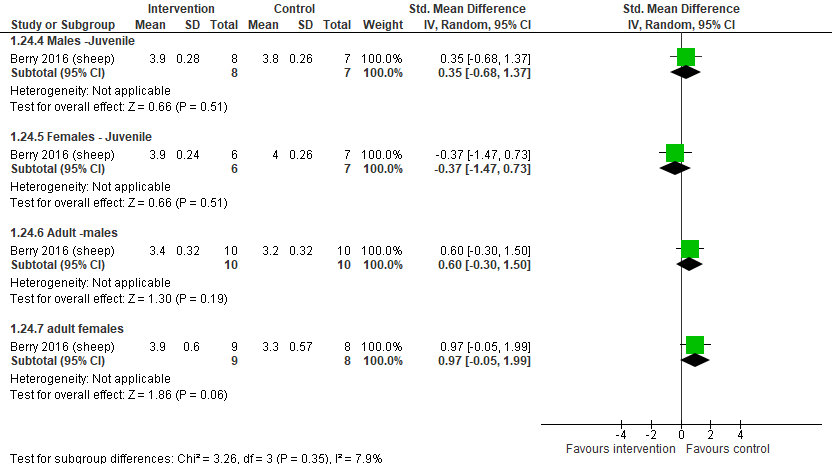


**Insulin sensitivity**

# d.


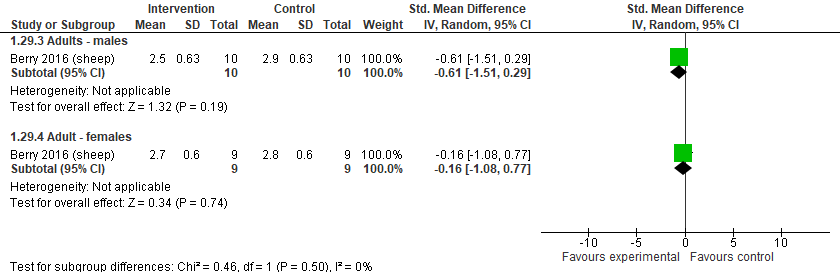


# e.


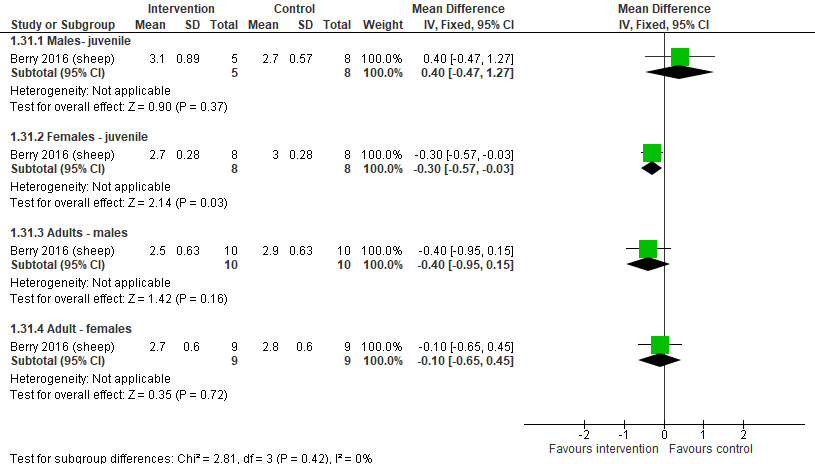


# Fasting insulin concentrations

# f.


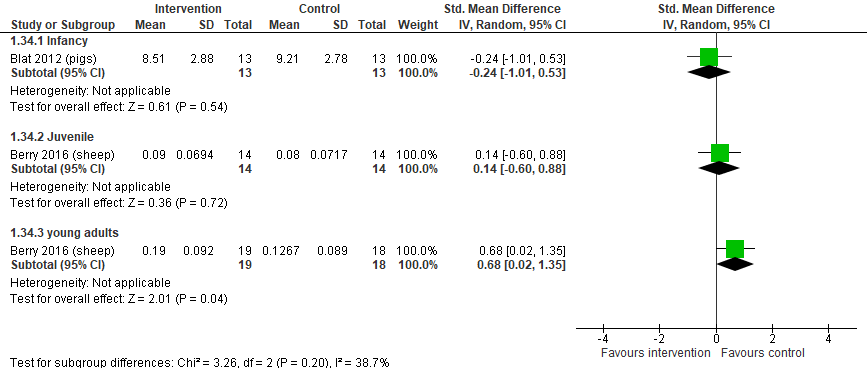


# g.


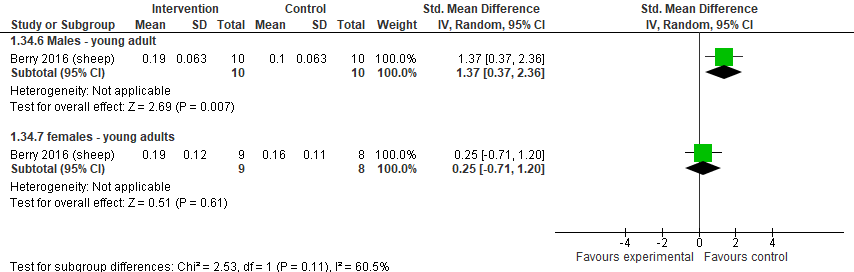


# h.


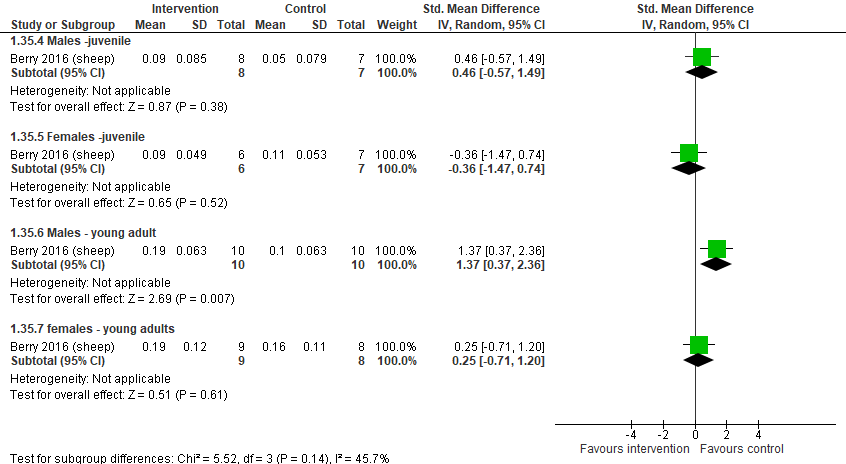


**Forest plots showing standardized mean differences and 95% confidence intervals**

# Supplementary Figure S4. The effect of macronutrient supplementation vs. no supplementation on a) overall energy intake b) overall appetite

#

# Overall Energy intake


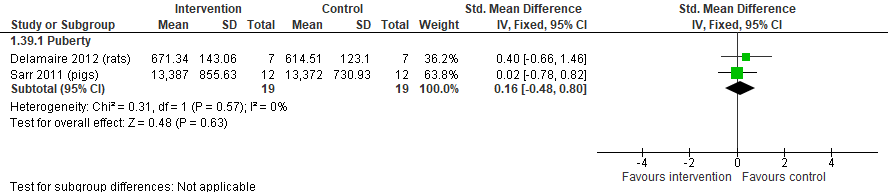


# Overall Appetite


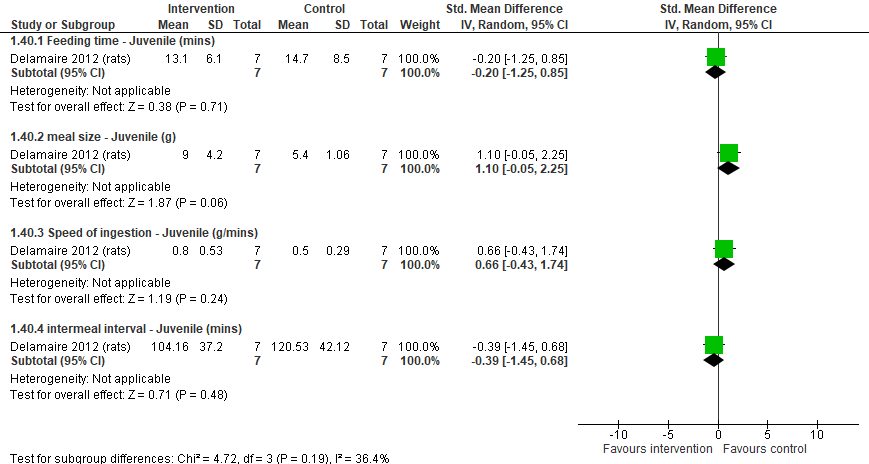


**Forest plots showing standardized mean differences and 95% confidence intervals**
